# Ion-Specific Modulation of the Conformation and Compactness of DNA Oligo-Catenanes

**DOI:** 10.1021/acs.jpcb.5c04107

**Published:** 2025-12-30

**Authors:** Terpsichori S. Alexiou, Christos N. Likos

**Affiliations:** Faculty of Physics, 27258 University of Vienna, Boltzmanngasse 5, Vienna 1090, Austria

## Abstract

The scope of the work presented here is the computational investigation of the effects of counterion valency, on the ensuing pairwise effective interactions between topologically interlinked DNA ring molecules within the dilute solution regime. Atomistic molecular dynamics (MD) simulations were performed for double stranded DNA [2]-catenanes in low to moderate ionic strength solutions of monovalent sodium and divalent counterions calcium and magnesium solutions. Upon catenation, stiff DNA minicircles obtain more anisotropic, more aspherical and slightly more oblate shapes, compared to their noncatenated conformations. In-plane ring stretching is observed in the catenated structures, which facilitates the spreading out of charges and reduces steric hindrance. Prominent effects of the ion type on the separation of the center-of-masses of the individual minicircles, as well as their preferred relative orientations have been established. An interesting interplay arises upon increasing ionic strength of divalent counterions, depending on the contour length of the individual DNA minicircle constituents: short DNA minicircles give rise to a nonmonotonic behavior that comprises sequential elongation and contraction regimes of the catenane, but longer minicircles give rise only to contracted catenane conformations.

## Graphical abstract

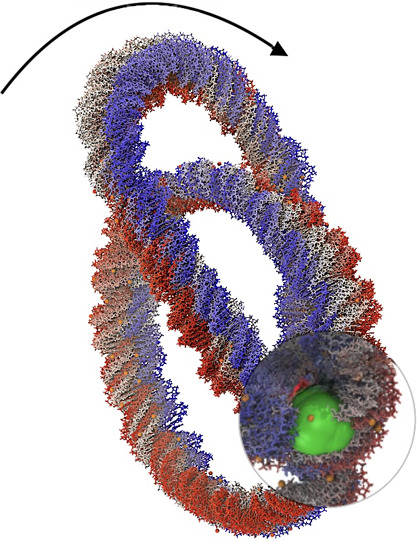

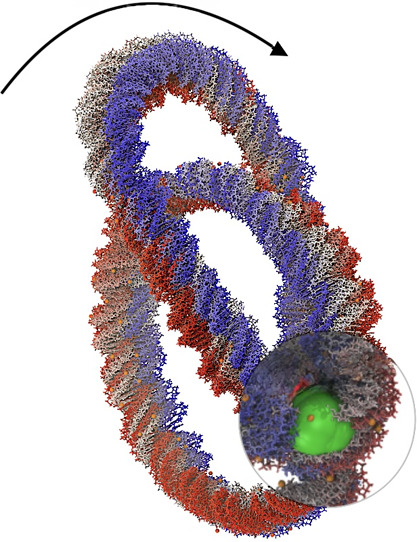

## 1 Introduction

Catenanes, an archetypal example of topologically intertwined molecules, comprise macrocycles threaded together akin to links in a chain, and are frequently encountered in diverse naturally occurring biological systems. Catenated DNA structures are involved in bacterial and viral DNA replication, where the newly synthesized DNA strands can form short-lived catenanes before being fully separated under the action of Topoisomerases.^1,2^ Another characteristic example of a structurally complex, catenated system is the kinetoplast DNA (kDNA),^3^ a unique mitochondrial structure that is common to unicellular flagellar organisms.

In addition to naturally occurring biological catenated polymers, synthetically produced DNA catenanes are now gaining increasing attention as promising materials with tunable configurations, by means of stimuli like pH and ions.^4^ Owing to advances in click chemistry,^5^ synthetic and enzymatic ligation techniques, the use of DNA for constructing these interlocked structures has given rise to applications like sensors, molecular machines and logic gates.^6^ Through a random library synthetic approach, the constituent minicircles can exhibit the capability of operating as independent units, that can facilitate functions such as DNA hybridization, and rolling circle amplification.^7^ A lot of research effort has also been devoted to the functionalization of DNA catenanes by the inclusion of single-stranded moieties with binding or a catalytic activity (e.g., DNAzymes or DNA aptamers),^8^ including unusual ssDNA motifs, such as G-quadruplexes and pH-responsive i-motifs, which can assume ion-dependent or pH-triggered folded states.^9–12^

Despite the important advancements achieved in the synthesis of DNA catenanes, experimental challenges still persist, particularly in synthesizing longer catenanes with a satisfactory yield, or in the precise characterization and efficient separation of the produced catenane products. Theoretical and computational models have been employed systematically in the past decade to advance the fundamental understanding of the physics of catenanes. The scaling of the mean radius of gyration of poly­[*n*]­catenanes-linear chains has been investigated as a function of the size, number and rigidity of linked rings.^13–17^ The stress relaxation of poly­[*n*]­catenanes is very fast and may be conveniently separated into ring-like and linear-like contributions, in agreement with the Rouse theoretical picture. However, viscosity has a surprising nonmonotonic dependence on the ring size in the long chain limit, contrary to theoretical predictions.^14,15,18,19^ Computational investigation of the response of catenane chains to mechanical stretching^13,20^ has revealed that the topological catenation makes [n]­catenanes exhibit larger elastic moduli than their linear and [*n*]­bonded-ring counterparts. In conditions of strong nanochannel confinement,^21^ the size and shape of the constituent rings of a catenane chain are greatly affected, and the rings acquire a uniaxial anisotropy. A comparison with isolated rings subjected to the same degree of confinement reveals that the mechanical bonding constraint enhances this anisotropy. The pore translocation signature of DNA catenanes has also been investigated,^22–24^ revealing a distinct topology-dependence of the translocation time. The presence of topological links between polymeric rings gives rise to an increase of its θ-temperature in comparison to isolated linear and ring chains.^16,25^

Overall, simulations offer an appealing alternative route for the detailed study of catenated DNA systems, but the majority of pertinent computational studies remain limited to simplified, generic coarse-grained systems and the important effects of ionic charges on the conformation, flexibility, and dynamic properties of DNA^26–29^ are not taken into account. Nonmonotonic effects of ionic strength on the translational diffusion coefficient of semiflexible DNA fragments, as well as unexpectedly high diffusion coefficients in general are among various effects that have been long established due to electrostatic coupling between the DNA and its counterions.^27,28^ Most recently, a marked decrease in the overall size of DNA kinetoplast networks has been established in response to increasing ionic strength from 5 to 200 mM, and this has been primarily attributed to the long-range electrostatic interactions that give rise to a change in effective DNA width.^29^

The scope of the work presented here is the computational investigation of the effects of counterion valency, on the conformations and ensuing bonding interactions between topologically interlinked DNA molecules within the dilute solution regime. The molecular lengths studied in this work are less than 200 bp and highly relevant to novel therapeutic applications of short DNA minicircles.^30^ Such short and stiff DNA minicircles are also increasingly being used in emerging DNA nanotechnological applications like functionalized origami and responsive catenanes,^31–33^ but their properties have so far received limited theoretical and computational attention.

The structure of the rest of the manuscript is as follows: in Section 2, we provide details of the simulated DNA systems, and the simulation protocol used to carry out molecular dynamics (MD) simulations. The effective interactions between the center-of-masses of the pairs of catenated DNA molecules, are presented in Section 3. Emphasis is placed on the investigation of the role of counterions and the mechanism of counterion condensation. Finally, in Section 4, we summarize the main conclusions and outlook of the present work and outline future plans.

## 2 Systems Studied and Simulation Details

The effects of low to moderate ionic strength solutions of monovalent (Na^+^) and divalent counterions (Mg^2+^, Ca^2+^) on the DNA catenane conformation and the effective interactions between the constituent minicircles are investigated by means of detailed atomistic molecular dynamics (MD) simulations. For the sake of simplicity and computational efficiency, the focus here is placed on [2]-catenanes. These are composed of double stranded, torsionally relaxed DNA minicircles with contour lengths of 65 bp and 180 bp that were generated using the nab module of AmberTools.^34^ The base sequences of the ds minicircles studied are provided in Table S1 of the Supporting Information. A vertically oriented pair of minicircle DNA molecules was placed within a cubic simulation cell in an interlocked configuration such that the planes of the two minicircles are approximately orthogonal and the normal vector of one lies within the plane of the other. All systems were solvated with the inclusion of SPC/E water molecules. Details of the properties of the systems investigated are provided in Table 1 and in the Supporting Information. For the neutralization of the DNA pair charges, an appropriate number of either sodium, calcium, or magnesium counterions was included, and then sufficient Na^+^Cl^–^, Ca^2+^Cl^–^, or Mg^2+^Cl^–^ ion pairs were added to achieve the range of bulk ionic strengths displayed in Table 1, using the Smith and Dang force field parameters^35^ for sodium and the Li and Merz force field parameters^36^ for calcium and magnesium. All ions were initially placed randomly at distances at least 5 Å from the solute and at least 3.5 Å from one another. The last generation general-purpose AMBER force field that takes into account the most recent parmbsc1 modifications^37^ introduced by the Barcelona Supercomputing Group to improve upon the parametrization of the backbone ε, ζ and glycosidic torsion angle χ dihedral angle force–field parameters were adopted for the DNA. Energy minimization, equilibration and MD simulation of the system of DNA pairs, ions, and water solvent molecules was performed using a standard multistage protocol.^38–41^ Following equilibration, production simulations were performed for up to 1 μs with a 2 fs time step. All MD production simulations were conducted in the isothermal–isobaric (NPT) statistical ensemble by making use of the Nosé–Hoover thermostat^42^ coupled with the Parrinello–Rahman^43^ barostat to maintain temperature *T* and pressure *P* fixed at their prescribed values of *T* = 300 K and *P* = 1 atm. The MD simulations were performed with the ΑΜΒΕΡ software.^34,44,45^ For each one of the 15 systems of catenated minicircles included in Table 1, independent simulations were also performed at the same ionic strength conditions, for the respective cases of single, noncatenated minicircles. This allows us to form a basis of comparison for the effects of catenation on the conformation of stiff DNA minicircles.

**Table 1:** Catenated Systems Studied and Simulated Conditions: System Acronyms in First Column Designate DNA Topology (R-), Number of bps (65, or 180), Type of Counterion Used (Na^+^, Ca^2+^, Mg^2+^), and Index Corresponding to the Respective Value of Increasing Ionic Strength in the Simulation Series (1,2,3), as Displayed in the Last Column

system	*N*_CΓ_ [-]	*C*_CΓ_ [mol/lt]	*N*_Cation^+^_ [-]	*C*_Cation^+^_ [mol/lt]	*C*_salt_ [mol/lt]	*I*_salt_ [mol/lt]
R65NaIs1	204	0.075	204	0.075	0.075	0.075
R65NaIs2	510	0.130	510	0.130	0.130	0.130
R65NaIs3	1020	0.260	1020	0.260	0.260	0.260
R65CaIs1	204	0.056	102	0.028	0.028	0.084
R65CaIs2	510	0.140	255	0.070	0.070	0.210
R65CaIs3	1020	0.280	510	0.140	0.140	0.420
R65MgIs1	204	0.058	102	0.029	0.029	0.087
R65MgIs2	510	0.150	255	0.073	0.073	0.219
R65MgIs3	1020	0.290	510	0.146	0.146	0.439
R180NaIs1	204	0.008	204	0.008	0.008	0.008
R180NaIs2	1020	0.041	1020	0.041	0.041	0.041
R180CaIs1	204	0.007	102	0.003	0.003	0.010
R180CaIs2	1020	0.041	510	0.021	0.021	0.062
R180MgIs1	204	0.008	102	0.004	0.004	0.013
R180MgIs2	1020	0.043	510	0.022	0.022	0.065

For each one of the 30 systems studied here (i.e, the15 catenated systems listed in Table 1 and their respective 15 single minicircle counterparts), a single simulation of long simulation time (1 μs) in total was performed. To ensure statistical rigor and reproducibility, five independent replicas were simulated for 4 selected systems out of the 30 in total studied here, namely for the catenated and single minicircle cases of the R65MgIs2 and R65MgCaIs2 systems listed in Table 1. The effects or number of replicas and simulation time on the estimated errors of the conformational properties and effective potentials for these systems are elaborated in the Supporting Information specifically in Figure S1 and Table S2.

## 3 Results and Discussion

### 3.1 Effects of Catenation on the Conformation of DNA Minicircles

Following the completion of the MD production runs, the global conformational properties of the catenated DNA rings have been analyzed. The radius of gyration tensor for each DNA molecule is calculated, as defined by the dyadic $S=\frac{1}{N}\sum_{i=0}^{n}r_{i}r_{i}^{T}$ with ***r***_*i*_ denoting the position vector of atom i in a frame of reference with origin set at the center-of-mass of the molecule. Upon transformation to a principal axis system that diagonalizes ***S*** such that ***S*** $=\left[\begin{array}{ccc} \lambda_{1}^{2} & 0 & 0\\ 0 & \lambda_{2}^{2} & 0\\ 0 & 0 & \lambda_{3}^{2} \end{array}\right]$ in this system, where the set {λ_1_^2^,λ_2_^2^,λ_3_^2^} denotes the eigenvalues (principal moments) of the tensor arranged in descending order, i.e., λ_1_^2^≥λ_2_^2^≥λ_3_^2^. The average size of each DNA minicircle can be estimated by computing the gyration radius *R*_g_ as the square root of the expectation value of the invariant *I*_1_ = λ_1_^2^+λ_2_^2^+λ_3_^2^: $R_{g}=\sqrt{{\left\langle R_{g}\right\rangle }^{2}=}\sqrt{I_{1}}$ . The three eigenvalues of the average radius-of gyration tensor provide a measure of the extent of the DNA minicircles along the corresponding three principal axes of this tensor. The first eigenvector corresponds to the axis of largest extension (major in-plane direction), with the second and third eigenvector correspond to the orthogonal in-plane axis, and the out-of-plane axis, respectively. Additional conformational shape measures are also computed, including

1 $$\left(a\right)\;the\;relative\;shape\;anisotropy,\kappa^{2},defined\;\text{as}:\kappa^{2}=1-3\frac{\left\langle \lambda_{1}^{2}\right\rangle \left\langle \lambda_{2}^{2}\right\rangle +\left\langle \lambda_{2}^{2}\right\rangle \left\langle \lambda_{3}^{2}\right\rangle +\left\langle \lambda_{3}^{2}\right\rangle \left\langle \lambda_{1}^{2}\right\rangle }{{\left(\left\langle \lambda_{1}^{2}\right\rangle +\left\langle \lambda_{2}^{2}\right\rangle +\left\langle \lambda_{3}^{2}\right\rangle \right)}^{2}}$$

2 $$\left(b\right)\;the\;asphericity\;b,\;defined\;\text{as}:b=\left\langle \lambda_{1}^{2}\right\rangle -\frac{1}{2}\left(\left\langle \lambda_{2}^{2}\right\rangle +\left\langle \lambda_{3}^{2}\right\rangle \right)$$

3 $$\left(c\right)\;the\;prolateness,S,defined\;\text{as}:S=\left\langle \frac{\left({3\lambda }_{1}^{2}-I_{1}\right)\left({3\lambda }_{2}^{2}-I_{1}\right)\left({3\lambda }_{3}^{2}-I_{1}\right)}{I_{1}^{3}}\right\rangle$$

The value of κ^2^ varies between 0 for highly symmetric conformations and 1 for a linear array of atoms, whereas b measures the deviation from the spherical symmetry. The prolateness values range from −0.25 to 2, with the negative values indicating oblate shapes, whereas the positive ones correspond to prolate objects.

The values of mean radius of gyration, the eigenvalues of the radius of gyration tensor, the relative shape anisotropy, and the apshericity are summarized in Tables 2 and 3, for the catenated and isolated systems studied here, respectively. Additionally, the values of the two characteristic ratios between the mean-squared eigenvalues, *r*_12_ = ⟨λ_1_^2^⟩/⟨λ_2_^2^⟩, and *r*_13_ = ⟨λ_1_^2^⟩/⟨λ_3_^2^⟩, are also of interest and are summarized in Figure 1.

**Table 2:** MD Estimates of the Mean Radius of Gyration $\left\langle R_{g}\right\rangle$ , the Eigenvalues of the Radius-Of-Gyration Tensor, <*λ*_1_^2^>,<*λ*_2_^2^>,<*λ*_3_^2^> the Relative Shape Anisotropy *k* ^2^, and Asphericity Parameter b for the Catenated Systems Studied Here

system	$\left\langle R_{g}\right\rangle$	<λ_1_^2^>	<λ_2_^2^>	<λ_3_^2^>	*k* ^2^	*b*
R65NaIs1	3.53 ± 0.05	7.35 ± 0.22	4.75 ± 0.35	0.29 ± 0.03	0.24 ± 0.05	4.82 ± 0.28
R65NaIs2	3.52 ± 0.10	7.43 ± 0.38	4.66 ± 0.66	0.29 ± 0.01	0.25 ± 0.08	4.94 ± 0.51
R65NaIs3	3.56 ± 0.06	7.29 ± 0.30	5.09 ± 0.33	0.29 ± 0.01	0.24 ± 0.04	4.60 ± 0.35
R65CaIs1	3.49 ± 0.08	6.93 ± 0.34	5.01 ± 0.44	0.29 ± 0.01	0.23 ± 0.00	4.28 ± 0.40
R65CaIs2	3.50 ± 0.07	7.92 ± 0.25	4.06 ± 0.47	0.30 ± 0.02	0.28 ± 0.00	5.73 ± 0.34
R65CaIs3	3.51 ± 0.07	7.59 ± 0.37	4.50 ± 0.40	0.29 ± 0.01	0.24 ± 0.03	5.20 ± 0.42
R65MgIs1	3.51 ± 0.08	7.22 ± 0.36	4.79 ± 0.47	0.29 ± 0.01	0.23 ± 0.06	4.68 ± 0.44
R65MgIs2	3.46 ± 0.08	7.72 ± 0.34	3.96 ± 0.46	0.31 ± 0.02	0.28 ± 0.08	5.58 ± 0.41
R65MgIs3	3.51 ± 0.08	7.39 ± 0.35	4.66 ± 0.47	0.29 ± 0.01	0.24 ± 0.06	4.91 ± 0.42
R180NaIs1	9.34 ± 0.09	48.14 ± 0.79	38.52 ± 1.41	0.62 ± 0.18	0.25 ± 0.02	28.55 ± 1.05
R180NaIs2	9.30 ± 0.16	48.52 ± 1.67	37.30 ± 2.49	0.65 ± 0.19	0.26 ± 0.03	29.52 ± 2.05
R180CaIs1	9.29 ± 0.30	51.86 ± 3.04	33.66 ± 4.52	0.81 ± 0.25	0.26 ± 0.08	34.55 ± 3.80
R180CaIs2	9.38 ± 0.18	49.51 ± 2.06	37.86 ± 2.56	0.58 ± 0.20	0.25 ± 0.03	30.28 ± 2.43
R180MgIs1	9.34 ± 0.09	47.51 ± 1.07	39.15 ± 1.26	0.69 ± 0.14	0.24 ± 0.02	27.58 ± 1.25
R180MgIs2	9.30 ± 0.42	50.68 ± 4.13	35.28 ± 6.63	0.71 ± 0.45	0.26 ± 0.10	32.68 ± 5.34

**Table 3:** MD Estimates of the Mean Radius of Gyration $\left\langle R_{g}\right\rangle$ , the Eigenvalues of the Radius-Of-Gyration Tensor <*λ*_1_^2^>,<*λ*_2_^2^>,<*λ*_3_^2^>, the Relative Shape Anisotropy *k* ^2^, and Asphericity Parameter b, for the Non-catenated Counterparts of the Systems Presented in Table 1

system	$\left\langle R_{g}\right\rangle$	<λ_1_^2^>	<λ_2_^2^>	<λ_3_^2^>	*k* ^2^	*b*
R65NaIs1	3.56 ± 0.09	7.00 ± 0.39	5.37 ± 0.50	0.29 ± 0.01	0.22 ± 0.04	4.17 ± 0.46
R65NaIs2	3.55 ± 0.02	6.60 ± 0.09	5.68 ± 0.14	0.30 ± 0.03	0.22 ± 0.02	3.61 ± 0.11
R65NaIs3	3.54 ± 0.04	6.69 ± 0.23	5.53 ± 0.10	0.30 ± 0.01	0.22 ± 0.02	3.77 ± 0.24
R65CaIs1	3.52 ± 0.04	6.69 ± 0.19	5.41 ± 0.24	0.30 ± 0.01	0.22 ± 0.02	3.84 ± 0.22
R65CaIs2	3.57 ± 0.05	6.95 ± 0.23	5.48 ± 0.26	0.29 ± 0.01	0.22 ± 0.02	4.07 ± 0.26
R65CaIs3	3.52 ± 0.15	7.52 ± 0.62	4.60 ± 0.83	0.28 ± 0.01	0.24 ± 0.11	5.08 ± 0.74
R65MgIs1	3.44 ± 0.12	7.98 ± 0.45	3.58 ± 0.70	0.28 ± 0.01	0.33 ± 0.12	6.05 ± 0.57
R65MgIs2	3.46 ± 0.06	6.78 ± 0.16	4.77 ± 0.38	0.40 ± 0.06	0.23 ± 0.05	–0.12 ± 0.03
R65MgIs3	3.53 ± 0.13	7.61 ± 0.51	4.58 ± 0.72	0.29 ± 0.00	0.26 ± 0.10	5.17 ± 0.62
R180NaIs1	9.38 ± 0.07	47.13 ± 0.79	40.29 ± 1.13	0.58 ± 0.29	0.24 ± 0.02	26.70 ± 0.97
R180NaIs2	9.32 ± 0.16	47.38 ± 2.08	38.97 ± 2.18	0.61 ± 0.36	0.25 ± 0.03	27.59 ± 2.35
R180CaIs1	9.38 ± 0.12	46.30 ± 1.25	41.26 ± 1.84	0.47 ± 0.10	0.24 ± 0.01	25.43 ± 1.54
R180CaIs2	9.40 ± 0.12	46.82 ± 1.06	41.07 ± 1.97	0.53 ± 0.13	0.24 ± 0.01	26.01 ± 1.44
R180MgIs1	9.37 ± 0.09	46.91 ± 0.78	40.27 ± 1.50	0.64 ± 0.19	0.24 ± 0.02	26.46 ± 1.08
R180MgIs2	9.37 ± 0.18	48.18 ± 2.05	39.00 ± 2.69	0.55 ± 0.15	0.25 ± 0.03	28.45 ± 2.44

**Figure 1:**
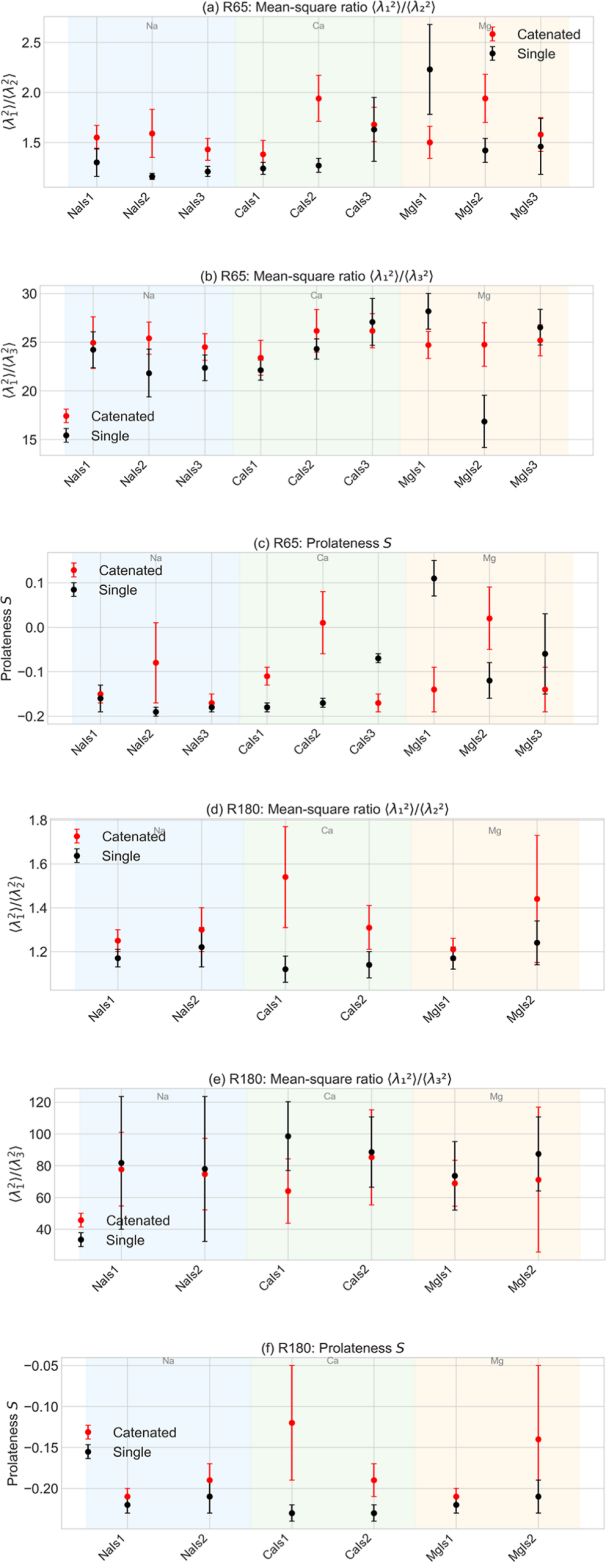
Effects of ion type and ionic strength on the characteristic ratios of the mean-squared eigenvalues, *r*12 = ⟨λ_1_^2^⟩/⟨λ_2_^2^⟩ and *r*13 = ⟨λ_1_^2^⟩/⟨λ_3_^2^⟩, as well as on the mean prolateness S of the systems studied here: scatter points indicate the respective values corresponding to either catenated or isolated rings, as indicated in the legend. Panels **(a**–**c)** correspond to 65 bp DNA minicircles and panels **(d**–**f)** to 180 bp DNA minicircles.

For all equilibrium averages of the conformational properties shown in Figure 1, Tables 2 and 3, the corresponding statistical errors have been estimated using the technique of block averaging for a single simulation of long simulation time, i.e. 1 μs in total. The errors estimated are quite small, thus the level of statistical noise can be deemed relatively low. Additional results from five independent replicas that were simulated for 4 selected systems, namely for the catenated and single minicircle cases of the R65MgIs2 and R65MgCaIs2 systems of Table 1, are shown in Table S2 and are in good agreement with the single long simulation results of Tables 2 and 3. Indicative time series plots of the eigenvalues of catenated rings are presented in Figures S2, S3.

It is interesting to assess how catenation influences DNA structure, by examining its effects on the conformation of individual minicircles within the linked systems as compared against their noncatenated state. The stiff minicircles studied here exhibit increased asphericity and anisotropy in their catenated state. This is further corroborated by the respective changes in the eigenvalues of the radius of gyration tensor: in the catenated state, a marked increase of the principal eigenvalue is evident, while the intermediate eigenvalue tends to be reduced, and the smallest one is only minimally affected by catenation. It appears that stiff rings redistribute their mass in plane by extending along their major principal axis so as to stretch their charges as far away as possible and reduce steric hindrance effects. This results in more pronounced elliptical conformations in the catenated state, characterized by increased ratio values, as shown in Figure 1. Out-of-plane bending fluctuations are restricted by the bending stiffness of these stiff DNA minicircles, therefore resulting in minimal changes in the smallest eigenvalue. Topological catenation imparts notable changes in the prolateness of rings: catenated rings are more oblate than rings in isolation. The effect is pronounced for shorter, stiffer rings: their catenated conformations appear to flatten and stretch in-plane, resembling stretched-out disk-like objects. This is associated with increased oblateness of the catenated structures. A notable exception occurs for the case of the short minicircles (65 bp) at the lowest ionic strength of magnesium ions studied here (0.087 M), where catenated structures exhibit more circular conformations in comparison to their isolated counterparts. This deviation can be related to the solvation structure and ionic condensation affinity of magnesium. It has been established that magnesium ions exhibit an extended, tight hydration layer and they tend to preferentially bind in the major groove region, rather than in the outer strand regions like calcium ions.^46,47^ Due to the fact that magnesium ions bind deep in the grooves, they constrain the DNA minicircle into more circular conformations. The fact that the binding of Mg­(2+) makes the DNA duplex more rigid compared to the sodium, has been demonstrated in previous studies by reduced conformational entropy and restricted local bending motion.^46^ In a similar fashion, for the case of the 180 bp minicircle in the higher ionic strength studied, the presence of magnesium bridges between minicircles also constrains their conformation to be more circular, as can also be seen from the respective prolateness value.

Previous simulation studies on flexible, uncharged ring polymers, have also demonstrated a similar effect of increased anisotropy, asphericity and oblateness in catenated ring polymer conformations.^7,48,49^ This is accompanied by a small degree of swelling of the rings (10% swelling in the limit of infinitely long rings). Due to the large bending stiffness of the DNA rings studied here, catenated rings incur negligible changes in their effective volume in comparison to their isolated state conformations. The effects of topological catenation on the characteristic ratios of the mean-squared eigenvalues, as well as on the mean prolateness of the rings for the systems studied here are presented in Figure 1, for the different types of ions and ionic strength values studied here.

### 3.2 Effective Interactions between Catenated Minicircles

In this section, the effective interaction between the centers of mass of the catenated DNA minicircles is calculated from the respective pair correlation function by calculating:, β*V*_eff_≡-ln­[*g*(*r*)] where β = 1/*k*_*B*_ *T*, and *g*(*r*) denotes the radial pair distribution function of the center of masses of the two catenated minicircles. The resulting effective pair interactions between the centers of mass of the constituent DNA minicircles of the [2]-catenanes studied here are shown in Figure 2.

**Figure 2:**
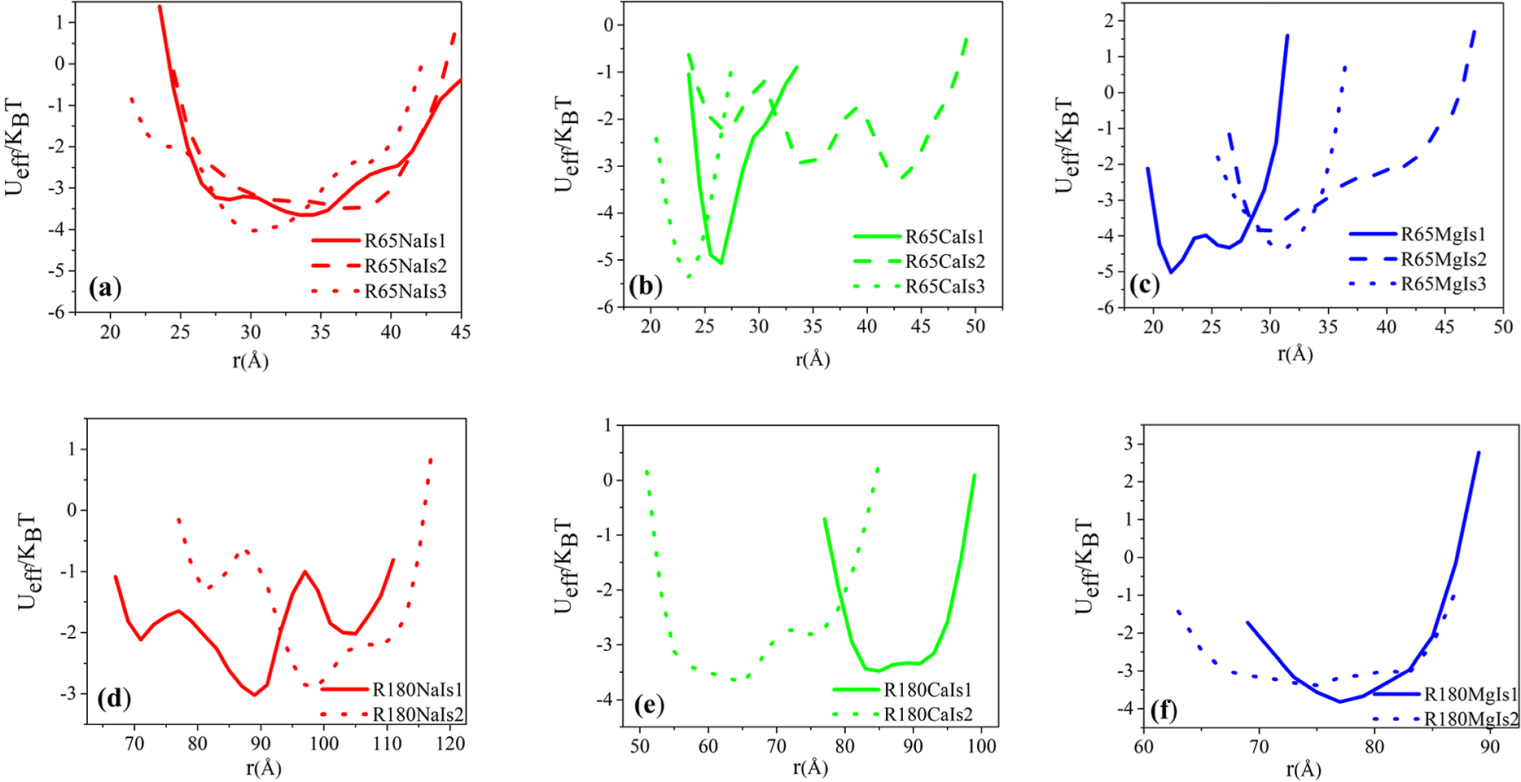
Effective isotropic potential between the centers of mass of a pair of linked ds-DNA minicircles: top and bottom rows correspond to a contour length of 65 bp and 180 bp, respectively. Panels (a–f) refer to systems with different ion type and ionic strength as denoted in legends.

In all cases studied, the qualitative shape of the effective potential resembles bell-shaved, convex form curves, as frequently encountered in several bonded type interactions. Two notable points deserve mention with respect to the effects of counterions: (a) the nonmonotonic dependence of the center-of-mass separation of the minicircle constituents of the [2]-catenane on ionic strength (see Figure 2a–c), and (b) the manifestation of double-well asymmetric potential forms (e.g., Figure 2b–d). An explanation of these seemingly surprising effects lies in the close observation of the catenated conformations that are adopted by DNA minicircles, as well as in the associated variations in the ionic atmosphere, and is exposed in more detail in the following sections.

The occurrence of multiple minima is evident in several catenated systems of this study: it is particularly pronounced for the 65 bp length catenated rings in the presence of both divalent ions, but it is also observed for longer rings of 180 bp length in the presence of monovalent sodium ions. In all these cases, the states corresponding to the primary minima can be distinguished by the fact that the catenated rings tend to prefer configurations that are more stretched–in-plane in comparison to the respective states correpsonding to the secondary minima. This is clearly indicated by the pertinent eigenvalue ratios presented in Table S5 of the Supporting Information. To ensure statistical rigor and reproducibility, two long time independent replicas (1 μs) were simulated for two selected systems, namely for the cases of the 65 bp length catenated rings in the intermediate ionic strength concentration of divalent ions (i.e., R65MgIs2 and R65MgCaIs2 systems listed in Table 1). The results for the effective potentials for these systems are calculated as the average across replicas and are presented in Figure S6 of the Supporting Information Good qualitative agreement is observed with the overall form of the multiwell potentials calculated from a single long simulation (Figure 2).

Upon increasing ionic strength, for the case of short minicircles of 65 bp length, an initial stretching phase occurs for intermediate ionic strength values. This is a direct consequence of ionic crowding, which becomes significant for shorter rings with smaller volume. Although the ionic strength rises in this low-to-intermediate ionic strength regime, the effective repulsion between rings is still partially screened, and the rings are being pushed further apart as the results of additional ions being constricted in the free volume between them. As the ionic strength is further increased to higher values, a compaction phase appears next: now the effective repulsion between DNA rings is fully screened and the electrostatic contribution no longer dominates the effective repulsion between rings. This allows the rings to approach closer and the catenanes obtain a contracted form, as shown in Figure 3.

**Figure 3:**
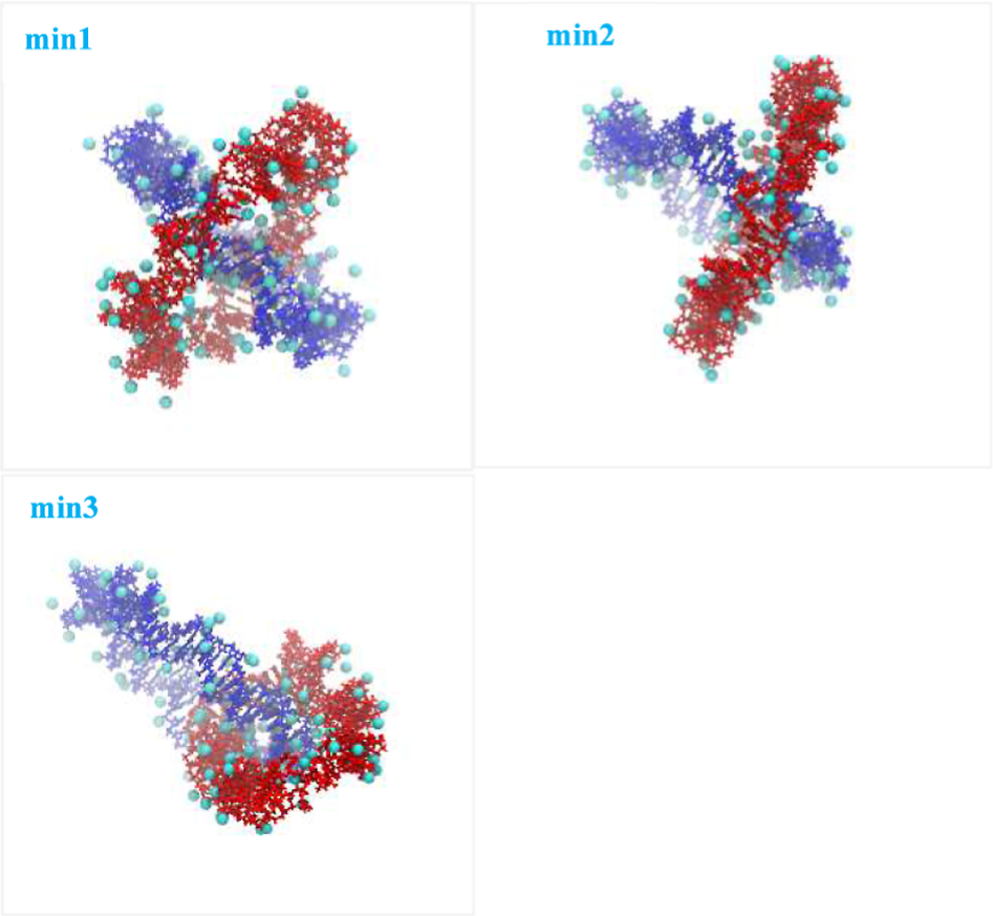
Indicative catenated conformations, each corresponding to a distinct instantaneous state that corresponds to each of the three minima regions of the effective interactions for system R65CaIs2, as shown in Figure 2c and Table S6. The minima are numbered in ascending center-of-mass separation distance, as noted in Table S6. The ionic atmosphere, composed of magnesium ions found within a 6.0 Å cutoff radius from the surface of DNA, is visualized in the form of beads.

As the size of the DNA rings increases to 180 bp, the ionic crowding effect no longer comes into play for the divalent ions, as it can been seen in Figure 2. Upon increasing ionic strength of calcium ions, DNA catenanes composed of 180 bp rings exhibit only contracted conformations. In this case, the screening effect imparted by the increased ionic strength allows the DNA rings to approach closer without any ionic crowding effects. In the case of magnesium ions, a broad plateau region appears for increased ionic strength, allowing the catenated rings to sample more contracted conformations. Only in the case of sodium ions, the catenanes keep extending upon increasing ionic strength due to the less effective screening effect of monovalent ions.

Another notable point is related to the formation of ionic bridges between the 180 bp long DNA rings in the case of the larger ionic strength of magnesium ions. Close contacts can form between the interlinked DNA rings in the presence of magnesium ions, as it can be seen in Figure 4, where selected conformations corresponding to different states belonging to the plateau region are visualized. Close contacts between the interlinked pair of DNA rings are found in a vicinity of tightly condensed magnesium ions, which allow for the formation of physical interlinks (anchor points) between the two topologically catenated rings. The formation of physical interlinks gives rise to a pivoting motion of the two catenated rings, where the relative rotational or angular movement of one ring with respect to the other, is constricted around the interlocked region (the linking point).

**Figure 4:**
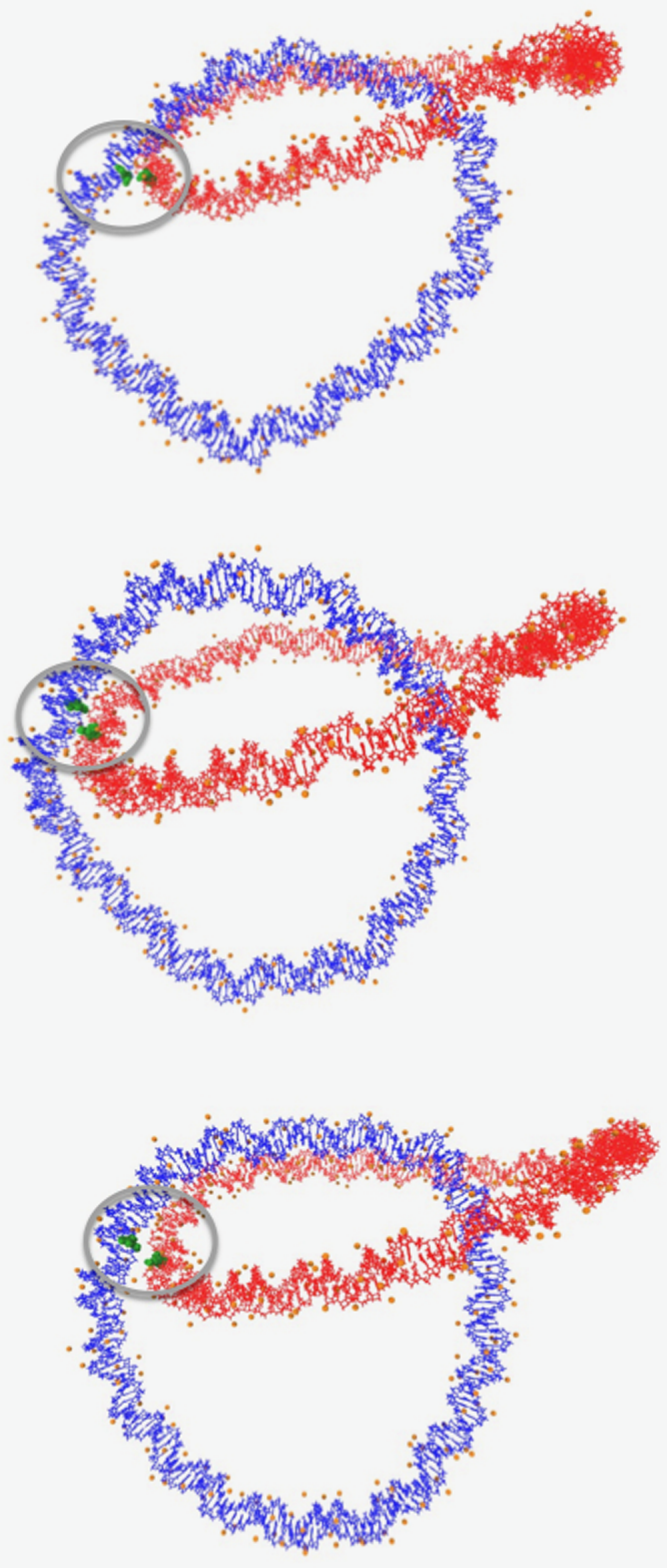
Indicative catenated conformations, each corresponding to a distinct instantaneous state that belongs to the plateau region of the effective interactions for system R180MgIs2 shown in Figure 2. The closest contact forming residues of the catenated minicircles, found within a 6.0 Å cutoff radius, are visualized in green and correspond to a specific pair of residues forming long lifetime contacts (region within gray circle). The ionic atmosphere, composed of magnesium ions found within a 6.0 Å cutoff radius from the surface of DNA, is visualized in the form of orange beads.

### 3.3 Pairwise Intermolecular Orientation Correlations

The relative angular orientation of the DNA molecule pairs is quantified in terms of the angle θ formed between the two eigenvectors ***p***_1_, ***p***_2_, corresponding to the largest eigenvalues of the two respective gyration tensors as follows.

4 $$\theta =\arccos\left(\frac{p_{1}\cdot p_{2}}{∥p_{1}∥∥p_{2}∥}\right)$$

The dependence of the estimated relative angular orientation angles on the center-of-mass distances is illustrated in Figures 4 and 5, for the cases of DNA minicircles of short (65 bp) and intermediate (180 bp) length. Results are shown in the form of probability density functions calculated from the respective scatter points. To this end, a 2D kernel density estimation (KDE) with a Gaussian kernel probability density function^50^ was employed in order to determine density values, as follows: $K\left(x\;,xˈ\right)=\exp\left(-\frac{{∥x-xˈ∥}^{2}}{2\sigma^{2}}\right)$ with ***x*** and ***x*** ^‘^denoting the corresponding vectors of values of a correlated pair of observables, namely the instantaneous average orientation angle and center-of-mass separation. The resulting population densities are visualized with different colors, by using a color map, as shown in Figures 5 and 6.

**Figure 5:**
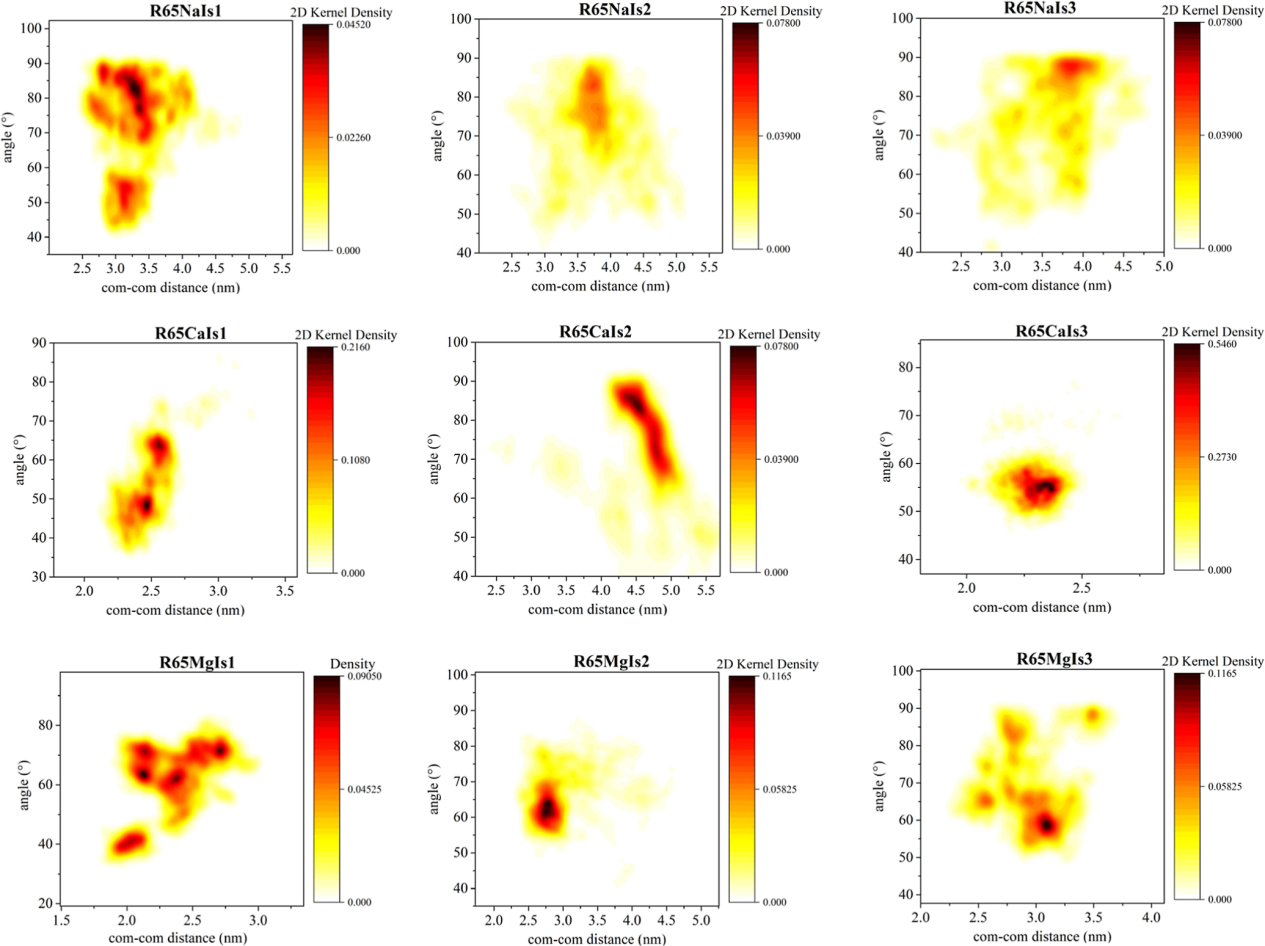
Probability density function of the average orientation angles between the eigenvectors of a pair of interlinked DNA molecules: case of 65 bp contour length DNA minicircles. Eigenvectors corresponding to the smallest eigenvalue of the respective gyration tensors are employed for the calculation.

**Figure 6:**
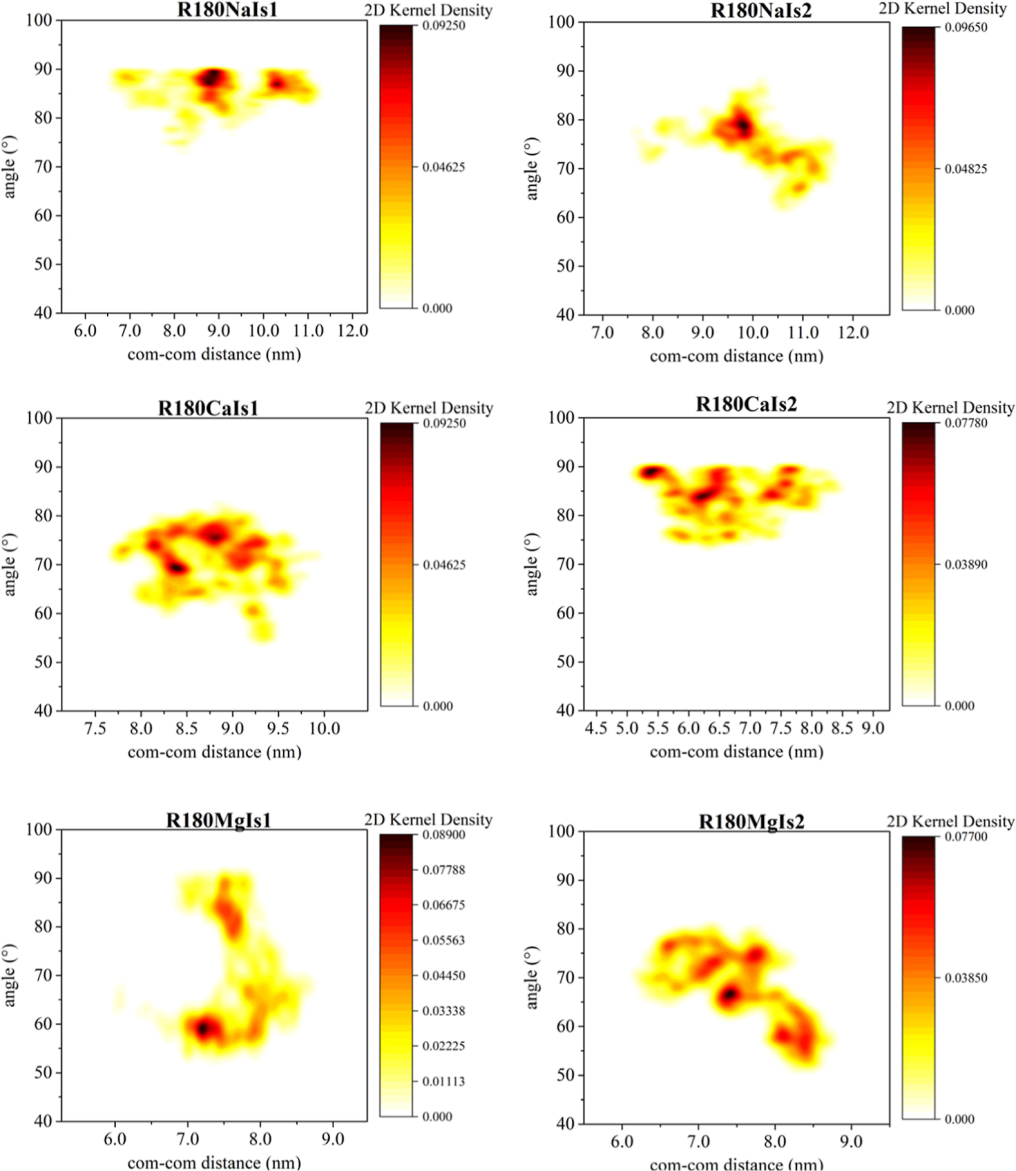
Probability density function of the average orientation angles between the eigenvectors of a pair of interlinked DNA molecules: case of 180 bp contour length DNA minicircles. Eigenvectors corresponding to the smallest eigenvalue of the respective gyration tensors are employed for the calculation.

Upon a close inspection of the correlations between the instantaneous relative orientation angle and center-of-mass separation for the case of linear DNA fragments, it becomes clear that short catenated rings maintain a practically vertical relative orientation in the presence of monovalent ions, over the entire ionic strength range studied. Only in the case of longer rings, can less orthogonal relative orientations be observed in sodium ionic solutions. This is made possible by the largest volume available for the relative movement of the catenated rings: rings can rotate and align closer without steric clashes as the contour length of the minicircles increases.

In ionic solutions of divalent ions, catenated rings with more aligned orientations are observed: the relative orientation angle between them in the presence of magnesium ions is in fact gradually reduced with increasing ionic strength, reaching values around 50. Due to the screening of electrostatic repulsion with increasing ionic strength, catenated rings can orient more parallel to each other, assuming conformations with larger surface area of each ring being in close proximity to the other ring. This trend is also evident in the case of longer rings (see Figure 6). In the case of calcium though, a nonmonotonic variation of the preferred relative orientation angle with ionic strength is observed, regardless of the contour length of the catenated rings. In particular, there is a regime where the relative orientation angle of catenated rings increases with increasing ionic strength of calcium chloride for both DNA ring lengths studied here. This can be related to the fact that for intermediate ionic strength values, the effective repulsion between the catenated rings is still partially screened and the approach of ring segments in a more aligned orientation is not energetically favorable. The two rings assume in fact larger relative orientation angles than the ones that correspond to smaller ionic strength of calcium chloride, due to either ionic crowding effects (prevalent for short rings) or due to the more diffuse screening effect of calcium, rising from preferential binding in the strand rather than the groove region.^47^ In ionic solutions of magnesium chloride on the other hand, due to groove binding preference of magnesium, stronger localized screening is effected and magnesium ions can form bridges that interlink the two rings. As a result, ring segments can approach closer in more tilted relative orientations, characterized by smaller relative orientation angles.

The relative orientations of catenated rings provide further support to our interpretation of the stretch-then-contract regime exhibited by shorter DNA minicircles in divalent ionic solutions. A detailed explanation is provided here. Based on the data in Table 2 and Figure 5 as well as on additional (unpublished) results. We refer here to the system R65Ca, in particular.

For the ideal case of a rigid, circular ring of radius *R*, the probability of finding a topological insertion perpendicular to the ring plane at distance *r* from any of its monomers is given by the expression

5 $$p\left(r\right)=\frac{r}{\pi R^{2}}\arccos\left(\frac{r}{2R}\right)$$

Accordingly, assuming two catenated, perfectly circular rings at right angles, the probability maximum for their centers of mass would be at about 1.5 *R*. As can be seen from Table 2 and Figure 5 for the lowest ionic strength (R65CaIs1) the distance is smaller than that and the rings do not stand perpendicular to each other. This deviation can only arise from the electrostatic interactions: as they are weakly screened in this case and a distance of 1.5 *R* would bring parts of the rings quite close to each other, the rings reduce their repulsions be reducing this distance and tilting toward each other.

Upon an increase of the ionic strength, the DNA rings become deformed. Indeed, as the electrostatic interactions become screened, it costs less energy for each ring to assume an ellipsoidal shape that brings two of its strands closer together, gaining thereby more room to place the centers of mass closer to the ideal distance mentioned above and assuming a more perpendicular orientation, as it happens for the R65CaIs2-system. A yet further increase of the ionic strength to the R65CaIs3-value crowds now also the inter-ring space with counterions, so that the rings cannot maintain a close proximity between their sharply curved segments and they are now pushed back into a conformation with a smaller distance between their centers of mass and a nonperpendicular orientation.

### 3.4 Ionic Atmosphere

In this section, the ionic atmosphere surrounding DNA is investigated by computing the radial pair distribution function g_ij_(*r*) of sodium, calcium, and magnesium counterions relative to selected DNA backbone and groove atoms, in order to determine preferential binding sites and facilitate the understanding of the effects of counterion correlations on the effective pair interactions of the catenated DNA molecules. To this end, the radial pair distribution functions of counterions relative to the phosphate group oxygen atoms, jointly denoted here as OP atoms, are computed, and typical results for magnesium ions are presented in Figures 7 and 8 of the main text, while Figures S4–S7 of the Supporting Information display results for sodium and calcium counterions. In a similar fashion, for the quantification of the ionic atmosphere in the DNA grooves, MD simulation results for the radial pair distribution function between sodium and calcium counterions and major and minor groove oxygen atoms are also computed and depicted in Figures S4–S7 of the Supporting Information, while Figures 7 and 8 presents results for magnesium ions.

**Figure 7:**
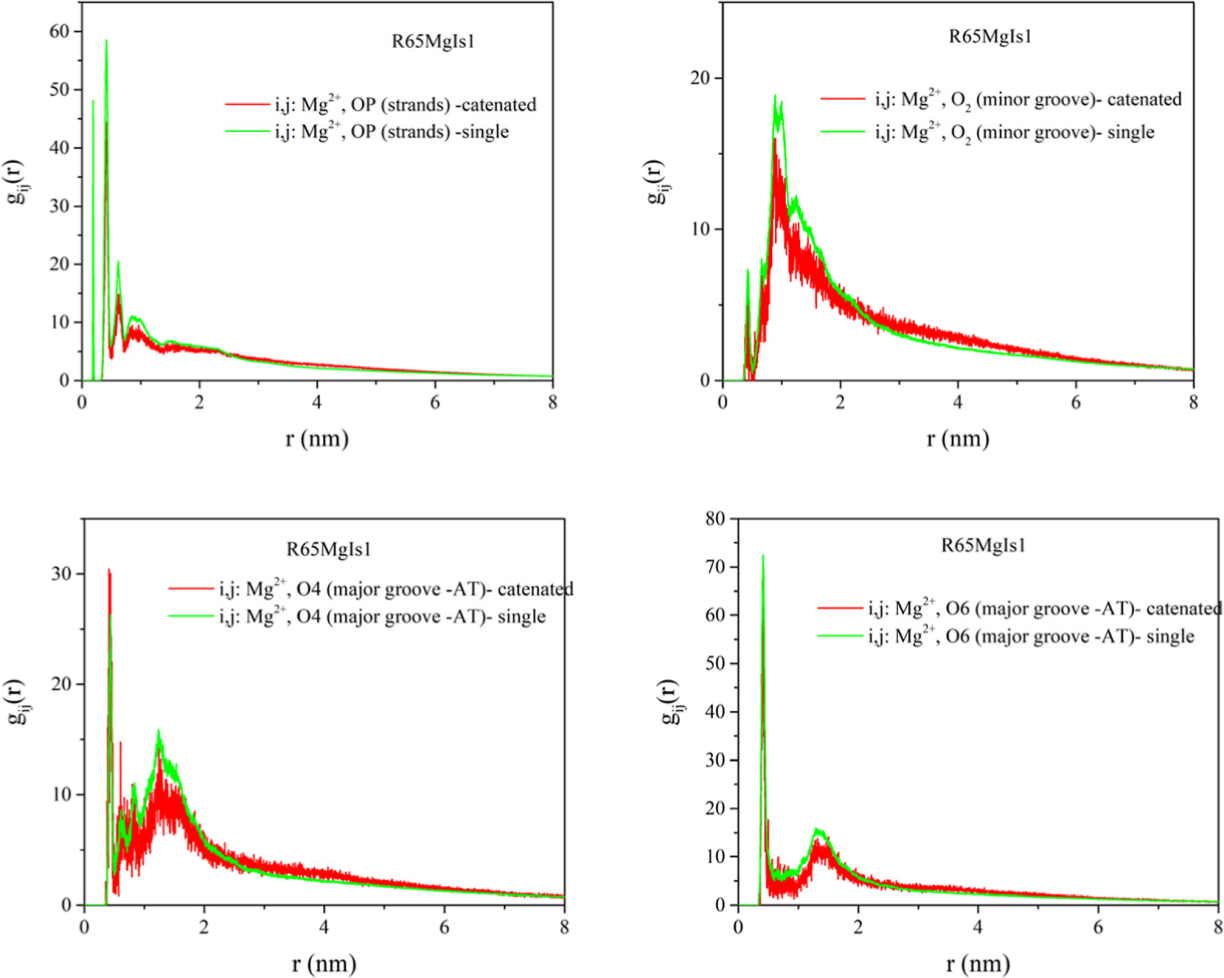
MD simulation results for the radial pair distribution function between Mg^2+^ salt counterions and: phosphate group oxygens OP (results have been averaged for the O1P and O2P type atoms following the amber force field naming convention), O2 minor groove atoms, O4 and O6 major groove atoms. Results shown correspond to the R65MgIs1 system of Table 1 in the manuscript. Results for both catenated systems (red line) and single minircircles (green line) are shown.

**Figure 8:**
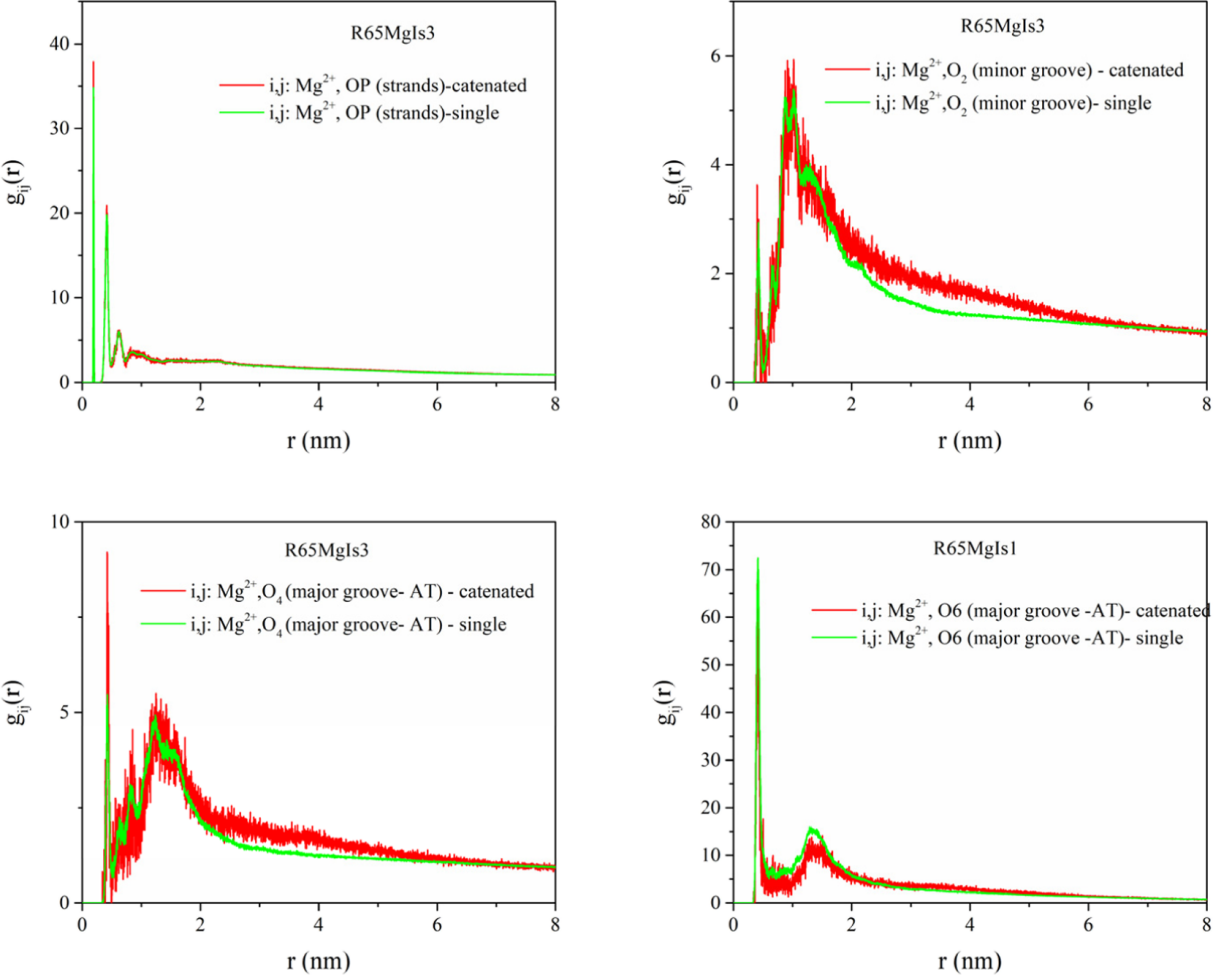
MD simulation results for the radial pair distribution function between Mg^2+^ salt counterions and: phosphate group oxygens OP (results have been averaged for the O1P and O2P type atoms following the amber force field naming convention), O2 minor groove atoms, O4 and O6 major groove atoms. The results shown correspond to the R65MgIs3 system of Table 1 in the manuscript. Results for both catenated systems (red line) and single minircircles (green line) are shown.

Distinct binding modes are evident in the condensation of each counterion type studied, with the monovalent sodium ions exhibiting a strong preferential binding in the vicinity of the DNA grooves, while the calcium ions are less frequently found within the groove region and are mostly located closer to the DNA strands. This finding is in close agreement with our previous work on the effective interactions between noncatenated DNA rings.^41^ Previous experimental studies point out that the alkali metal ions, including Na and K, are mostly located in the minor groove of AT-rich sequences, whereas divalent cations preferentially bind in the major groove of CG-rich base sequences.^51–54^ Additionally, theoretical studies suggest that the electrostatic potential of the minor groove of an AT base pair step is most negative, very closely followed by the GC major groove, the GC minor groove and, finally, the AT major groove.^55^ Thus, the strong binding preference of sodium for the major groove of the DNA minicircles that is observed herein could possibly be related to this base specific electronegativity ordering suggested by theoretical models.

For the case of magnesium cations, strong ionic condensation in the vicinity of the OP atoms is evident by the presence of intense peaks at a distance of approximately 0.16 nm. This closest contact distance between the DNA strand backbone and magnesium cations indicates direct magnesium-DNA interactions. Secondary peaks that occur at larger distances (e.g., ∼0.39 nm for the gij of magnesium and phosphate oxygen pairs), as well primary peaks of the radial pair distribution functions around minor and major groove atoms (∼0.43 nm for the case of O2 atom, ∼0.39 nm for the case of the O6 major groove atom) indicate water-mediated ion-DNA interactions.

In the entire range of ionic strength studied here, magnesium shows preferential binding in the major groove and phosphate (strand) regions, as compared to the minor groove region, on the basis of the intensity of the radial distribution function peaks. This preference has a steric origin: due to its extended hydration shell, Mg^2+^ cannot easily penetrate into the narrow minor groove region. Instead, Mg^2+^ ions can be more frequently located near the major groove, where they selectively bind to GC-base pair steps, which is consistent with the patterns found in X-ray crystal structures of divalent ion in B-DNA. ^47^The base-specificity of the binding of magnesium ions in the major groove has an electrostatic and not steric origin, as the CG major groove is more electrostatic than the AT major groove, despite being more narrow.^46,56^

In should be additionally noted that hydrated Mg^2+^ ions interact with DNA mainly through hydrogen bond interactions which are sensitive to the environment. As shown by previous studies,^46^ guanine bases can hold the hydrated Mg^2+^ ions more stably because adjacent N7 and O6 atoms can function as hydrogen bond acceptor and form hydrogen bonds simultaneously with one Mg^2+^ hexahydrated complex. On the contrary, the AT base pair does not provide a good electrostatic environment for hydrated Mg^2+^: the two hydrogen acceptors available, N7 atom of adenine and O4 atom of thymine, are separated by the hydrogen bond donor, namely the NH2 group on the adenine; this special configuration inhibits Mg^2+^ hydrated complexes from forming stable hydrogen bonds.^47^

Upon an increase in ionic strength, a more pronounced Mg^2+^ ion interaction with the phosphate group is evident, from the intensity of the respective radial distribution function. Increasing the ionic strength of MgCl_2_ enhances Mg^2+^ binding to the DNA backbone without significantly altering its binding preference toward the grooves.^46^ Mg condensates primarily at the major groove of CG base pair steps, in the vicinity of O6 type oxygen atoms, as suggested by the pair distribution functions in Figures 7 and 8. This is close agreement to previous studies.^57^

## 4 Conclusions

The effects of counterion valency on the resulting pairwise effective interactions between moderately short, stiff DNA minicircles in interlinked state have been computationally investigated by means of molecular dynamics simulations. Compared to their unlinked state, catenated minicircles assume conformations that are more anisotropic, more planar and increasingly stretched-in-plane, so as to minimize electrostatic and steric interactions. Similar to flexible, uncharged catenated ring polymers, catenated DNA minicircles tend to become more oblate upon catenation, but due to their high bending stiffness, there is no catenation-induced swelling effect.

Upon increasing ionic strength, an interesting, nonmonotonic variation of the center-of-mass separation of the catenaned rings has been established, giving rise to consecutive “stretch-then-compact” regimes. This implies that the catenated structure keeps on stretching up to a certain ionic strength but then starts compacting on further increase thereafter. An explanation for this nonintuitive effect is due to an interplay of ionic crowding effects and partially screened intramolecular repulsion: the crowded ionic milieu is not fully screening the effective repulsion between DNA rings in intermediate ionic strength values: electrostatic repulsion dominates in this regime, pushing the DNA rings further apart. From a critical ionic strength value onward, electrostatic repulsion is entirely screened, allowing the catenane components to approach closer. Ionic conditions thus appear to modulate the balance between electrostatic, steric and topological contributions to the effective repulsion, as well as the distance at which repulsion becomes negligible. This is of key importance for understanding stiff DNA ring behavior in solutions of varying salt content.

In the ionic milieu of magnesium, long-lifetime ionic bridges can form between the minicircle constituents, thus creating a physical interlink between them. The ion-induced interlink functions as an anchor point, that stiffens the structure locally and constrains the relative movement of the DNA minicircles in a pivoting motion pattern.

The potential implications arising from the findings of this study are 2-fold: based on the distinct ability of short minicircles to form catenanes that can stretch and compact in response to increasing ionic strength, novel chemical-stimuli-responsive structural transformations of DNA catenanes can be envisioned. In addition, on the basis of the formation of ionic bridges between the catenane constituents, an expanded range of mechanical properties of DNA catenanes can be anticipated.

Contrary to previous studies that focused on the intuitively expected monotonic compaction of DNA catenane networks in response to increasing ionic strength,^29^ this study establishes a nonmonotonic behavior that comprises sequential elongation and contraction regimes of an oligo-catenane composed of short and stiff DNA minicircles, whereas longer minicircles give rise only to contracted DNA conformations. Chemical-stimuli-responsive reversible structural transformations of DNA catenanes can offer unique functional advantages, such as efficient packaging with on-demand unfolding, which is ideal for drug delivery, gene therapy or DNA origami applications.

Furthermore, the formation of magnesium-induced intraring anchor points could hold significance for the enhancement of the force-bearing and energy-dissipation properties of catenanes. While individual Mg^2+^ bridges are noncovalent and reversible, their collective effect can significantly influence DNA structure and behavior. They can stabilize DNA–DNA interactions, promote compactness, and effectively modulate the mechanical properties of the corresponding catenane material through their dissociation and reassociation to dissipate energy, similar to other metal-coordinated catenane materials.^58^ The mechanical properties of such metal-coordinated DNA catenanes constitute an area of great practical and theoretical importance where our future work is directed to.

## Supplementary Material



## Notes

Information on base sequences used in simulations. Replica simulation results for conformational properties and effective potentials. Analysis of the ionic atmosphere and formation of contact points between catenated minicircles (PDF)
