# Supplementary material for: Ion-Specific Modulation of the Conformation and Compactness of DNA Oligo-Catenanes
Source: J Phys Chem B. 2025 Dec 30;130(2):868–80. doi: 10.1021/acs.jpcb.5c04107 (PMC12814548; doi:10.1021/acs.jpcb.5c04107)
Supplement: Supplementary file 1 [file jp5c04107_si_001.pdf]

Supporting Information for

Ion-Specific Modulation of the Conformation and Compactness of DNA oligo-Catenanes

Terpsichori S. Alexiou, [*†] *Christos N. Likos*

[†] Faculty of Physics, University of Vienna, Boltzmanngasse 5, 1090 Vienna, Austria

S1. Systems Studied and Simulation details

Table S1. Base sequences studied.

System	Base sequence
R65	d(ATCTT TGCGG CAGTT AATCG AACAA GACCC GTGCA ATGCT ATCGA CATCA AGGCC TATCG CTATT)
R180	d(ATCTT TGCGG CAGTT AATCG AACAA GACCC GTGCA ATGCT ATCGA CATCA AGGCC TATCG CTATT ACGGG GTTGG GAGTC AATGG GTTCA GGATG CAGGT GAGGA TATCT GTGCA ATGCT ATCGA CATCA AGGCC TATCG CTATT ACGGG GTTGG GAGTC AATGG GTTCA GGATG CAGGT GAGGA TATCT)

System	$<\lambda_1^2>$	$<\lambda_1^2>$	$<\lambda_1^2>$
R65CaIs2_cat	7.92±0.25	4.06±0.47	0.30 ± 0.02
R65MgIs2_cat	7.72±0.34	3.96±0.47	0.31±0.02
R65CaIs2_s	6.95±0.23	5.48±0.26	0.29±0.01
R65MgIs2_s	6.48±0.16	4.77±0.38	0.40±0.06

Table S2. MD Estimates of the quantities $\lambda_1, \lambda_2, \lambda_3$, for the catenated systems R65CaIs2 and R65MgIs2 of Table 2 and their respective non-catenated counterparts of Table3. Results are averaged across five independent replicas of simulation length ranging from 600ns – to 1μs.

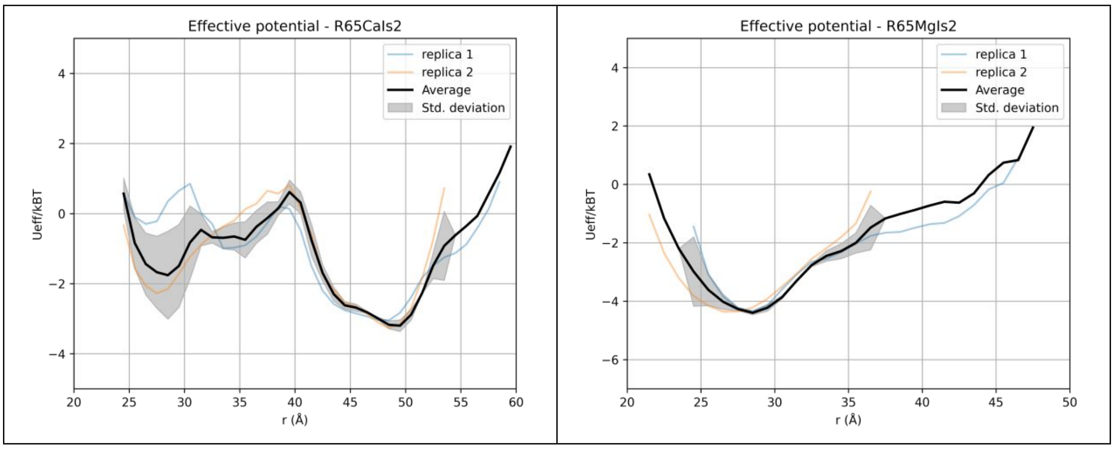

Figure S1. Effective potentials between the centers-of-mass of minicircle pairs for systems R65CaIs2 and R65MgIs2 of Table 1 in the main text. Results are shown for two long time replicas (1 μs). The average across replicas is also depicted.

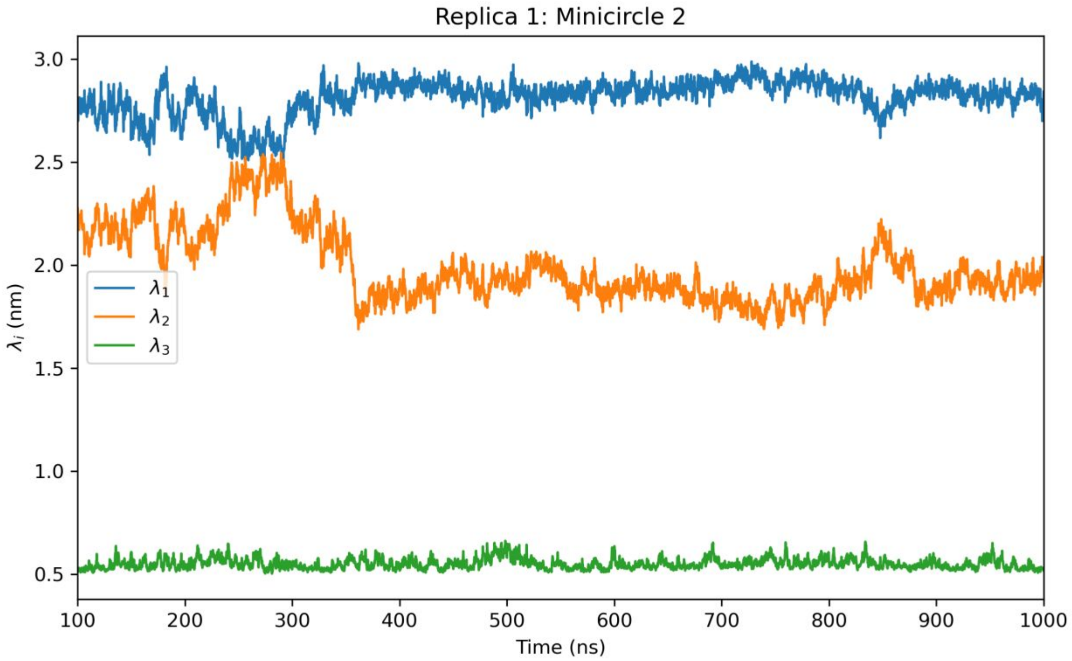

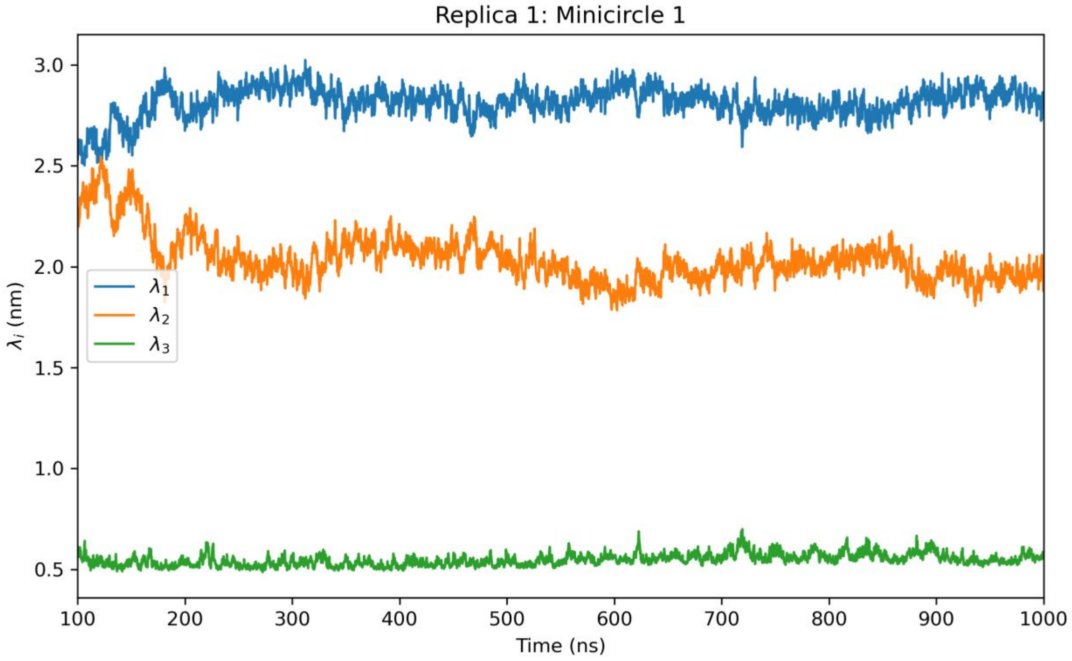

Figure S2. Time evolution of the eigenvalues (λ_1, λ_2, λ_3) of the gyration tensor for each DNA minicircle molecule for system R65CaIs2 of Table 1 in the main text.

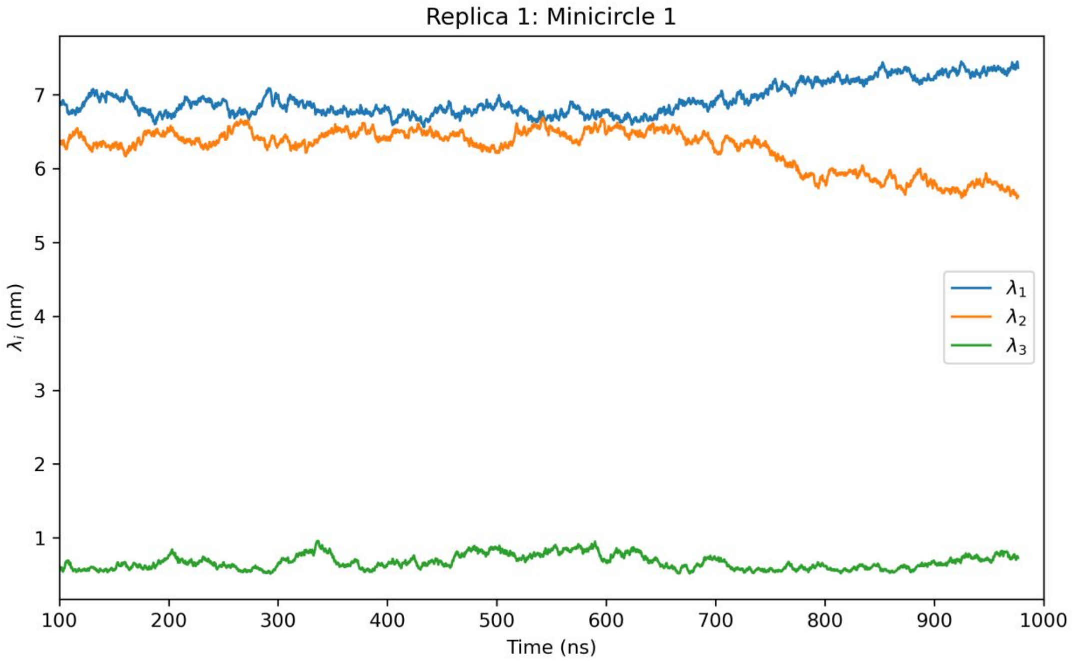

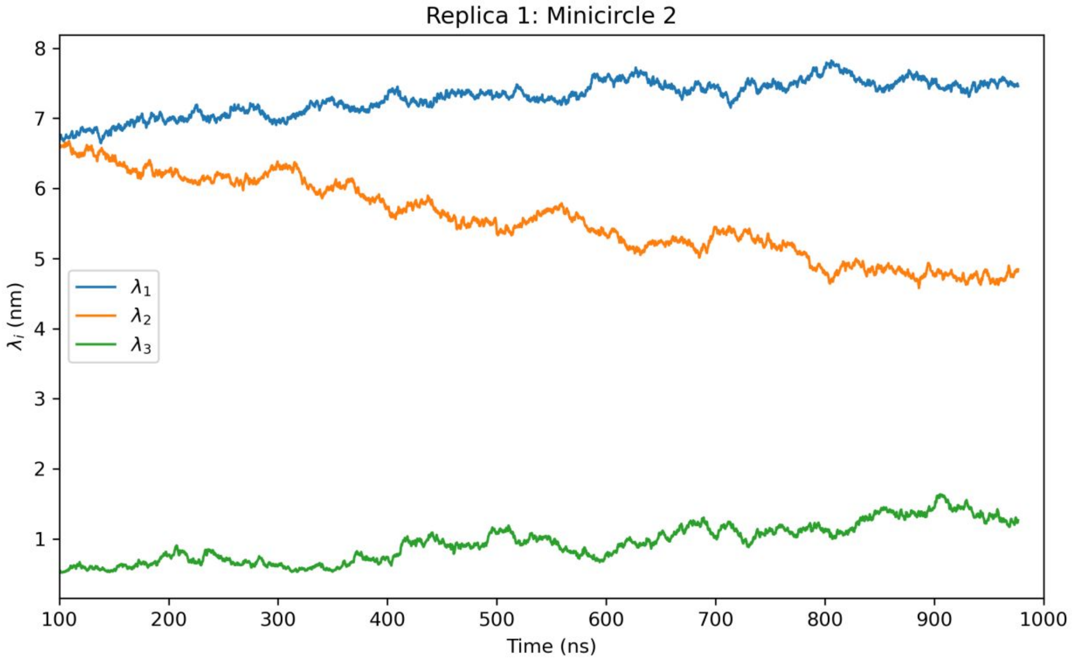

Figure S3. Time evolution of the eigenvalues (λ_1, λ_2, λ_3) of the gyration tensor for each DNA minicircle molecule for system R65180MgIs2 of Table 1 in the main text.

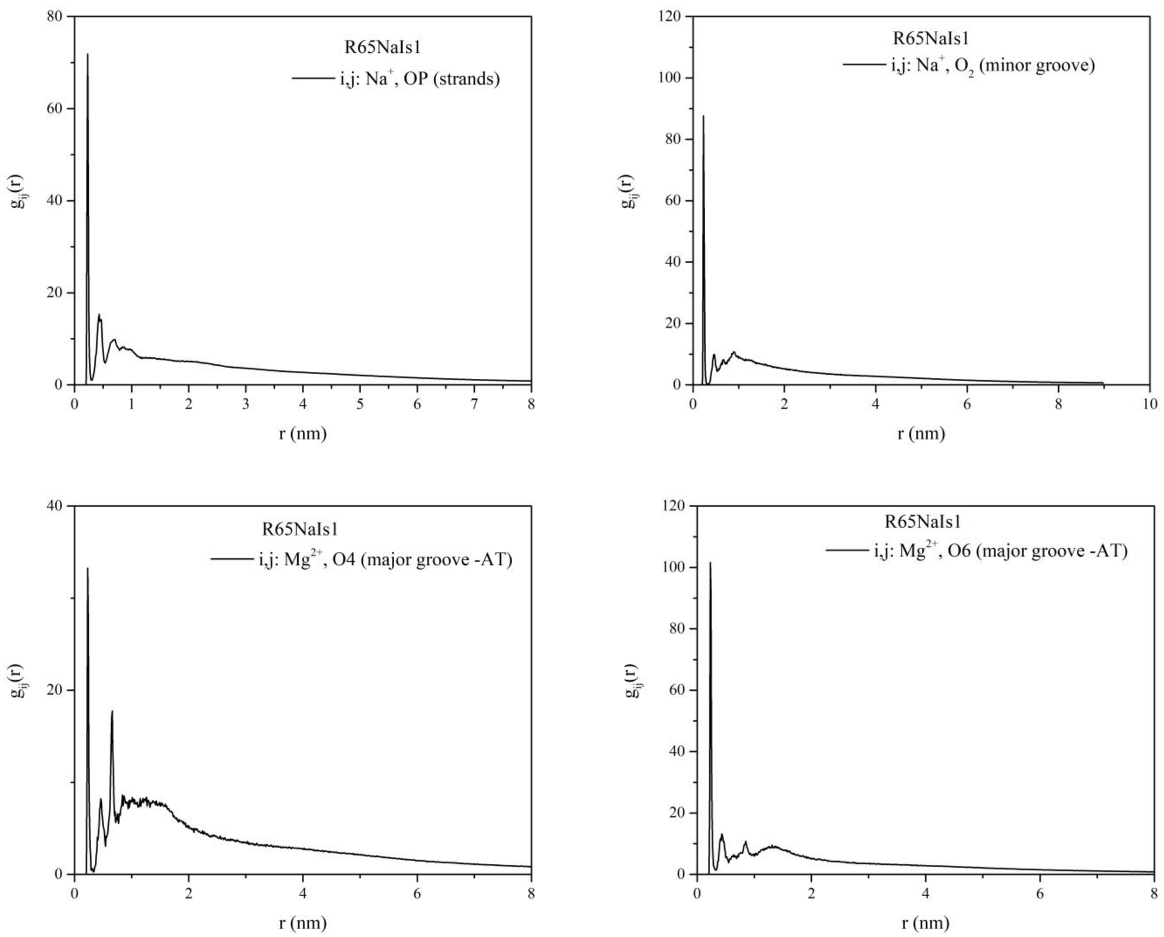

Figure S4. MD simulation results for the radial pair distribution function between Na+ salt counterions and: phosphate group oxygens OP (results have been averaged for the O1P and O2P type atoms following the amber force field naming convention), O2 minor groove atoms, O4 and O6 major groove atoms. Results shown correspond to the R65NaIs1 system of Table 1 in the manuscript.

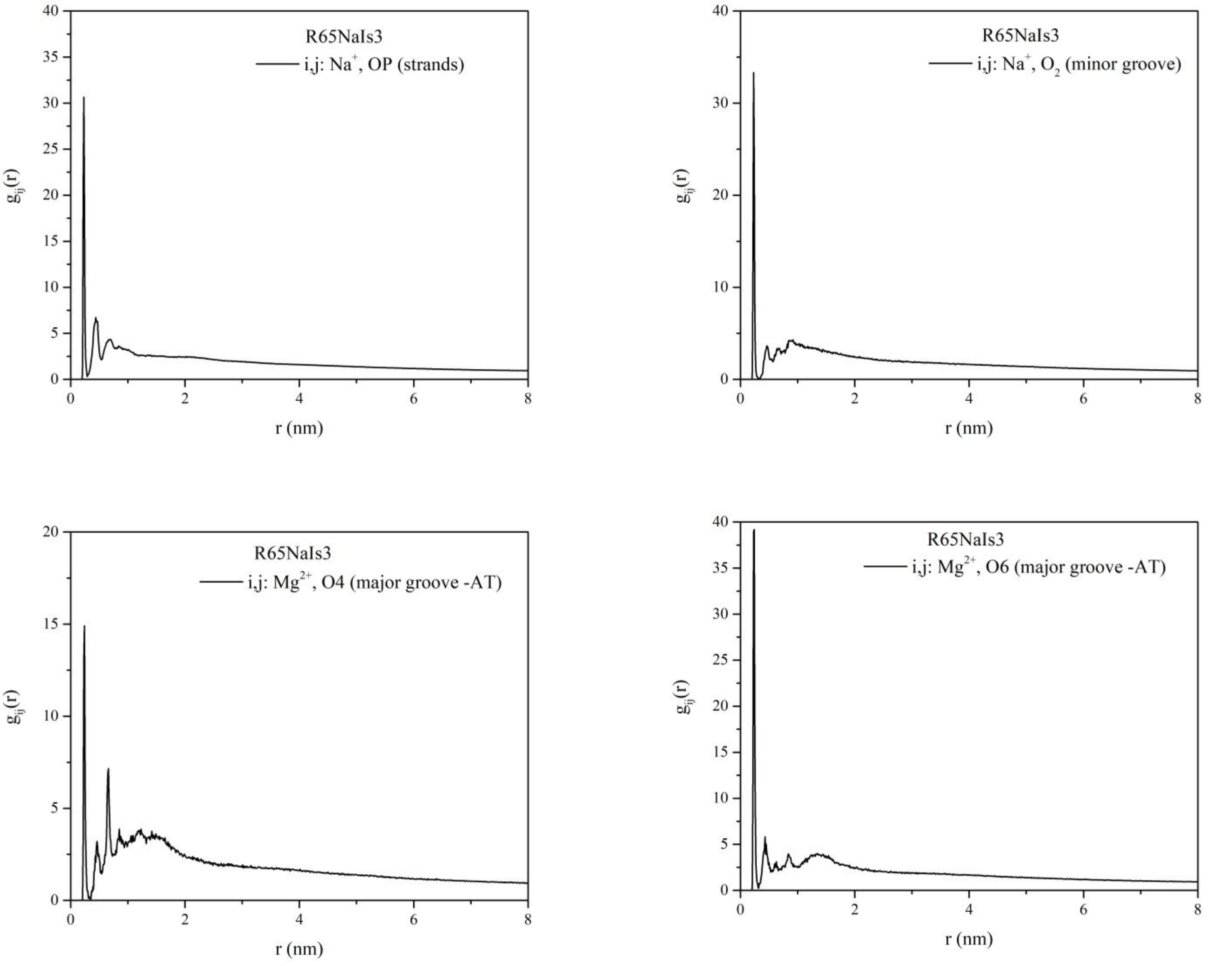

Figure S5. MD simulation results for the radial pair distribution function between Na+ salt counterions and: phosphate group oxygens OP (results have been averaged for the O1P and O2P type atoms following the amber force field naming convention), O2 minor groove atoms, O4 and O6 major groove atoms. Results shown correspond to the R65NaIs3 system of Table 1 in the manuscript.

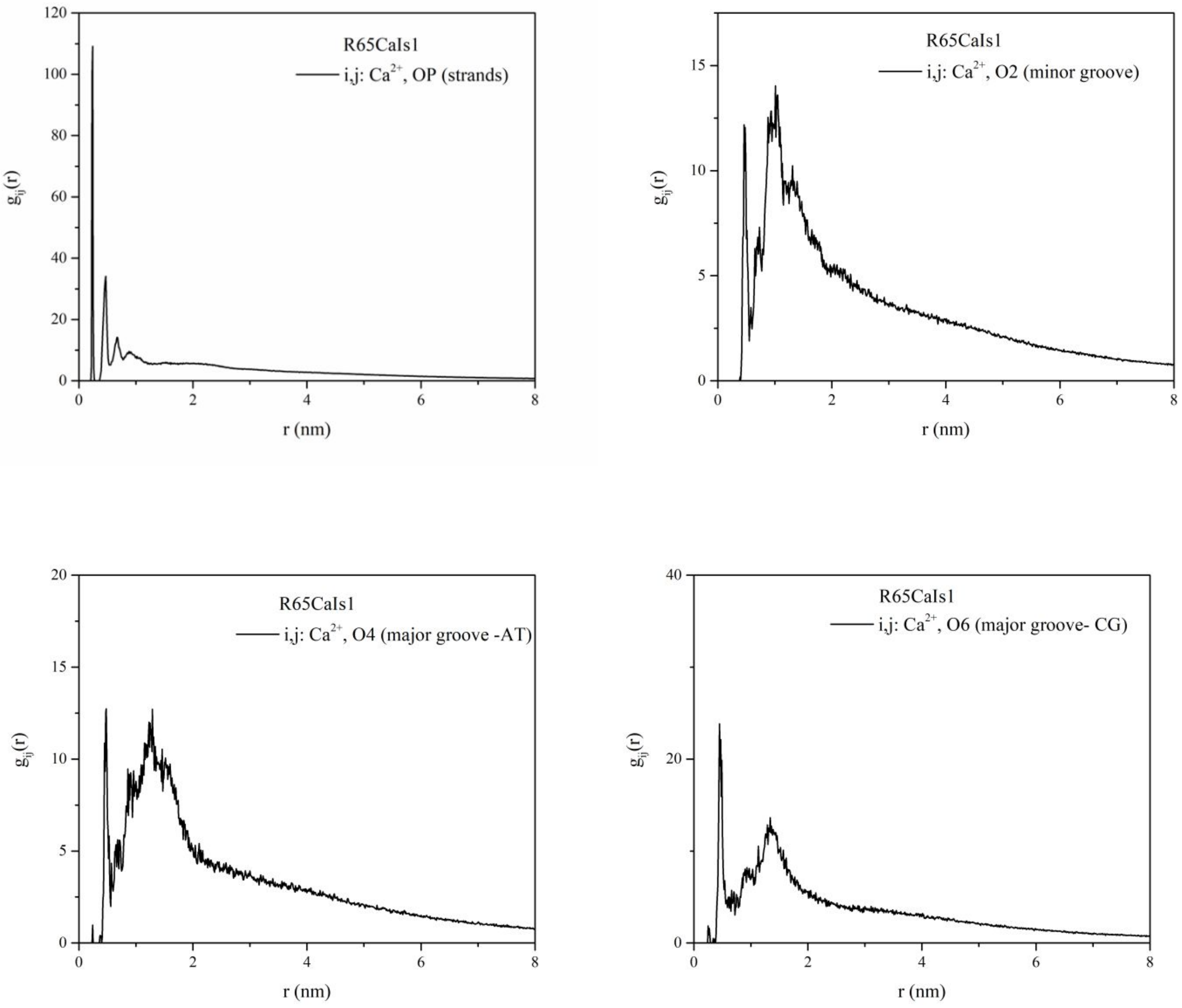

Figure S6. MD simulation results for the radial pair distribution function between Na+ salt counterions and: phosphate group oxygens OP (results have been averaged for the O1P and O2P type atoms following the amber force field naming convention), O2 minor groove atoms, O4 and O6 major groove atoms. Results shown correspond to the R65CaIs1 system of Table 1 in the manuscript.

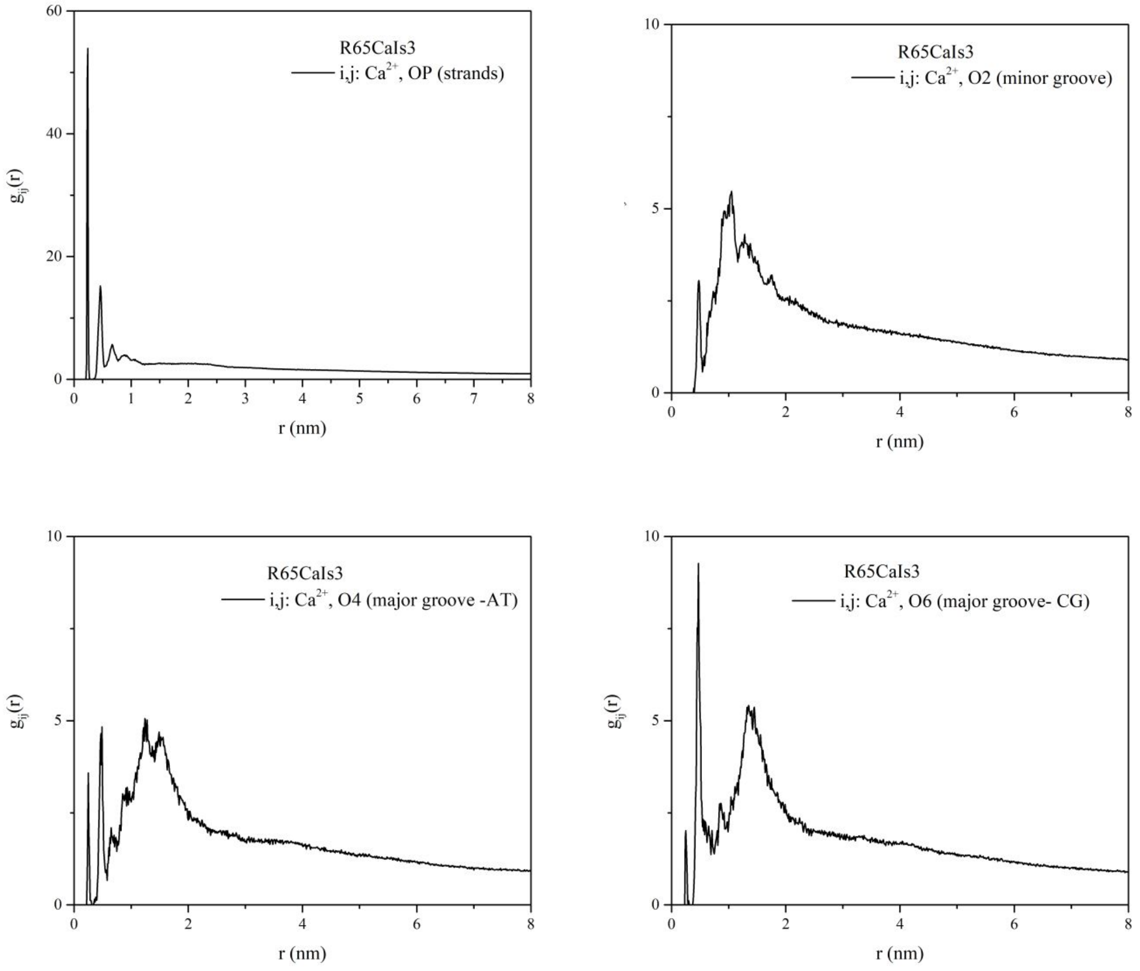

Figure S7. MD simulation results for the radial pair distribution function between Na+ salt counterions and: phosphate group oxygens OP (results have been averaged for the O1P and O2P type atoms following the amber force field naming convention), O2 minor groove atoms, O4 and O6 major groove atoms. Results shown correspond to the R65CaIs3 system of Table 1 in the manuscript.

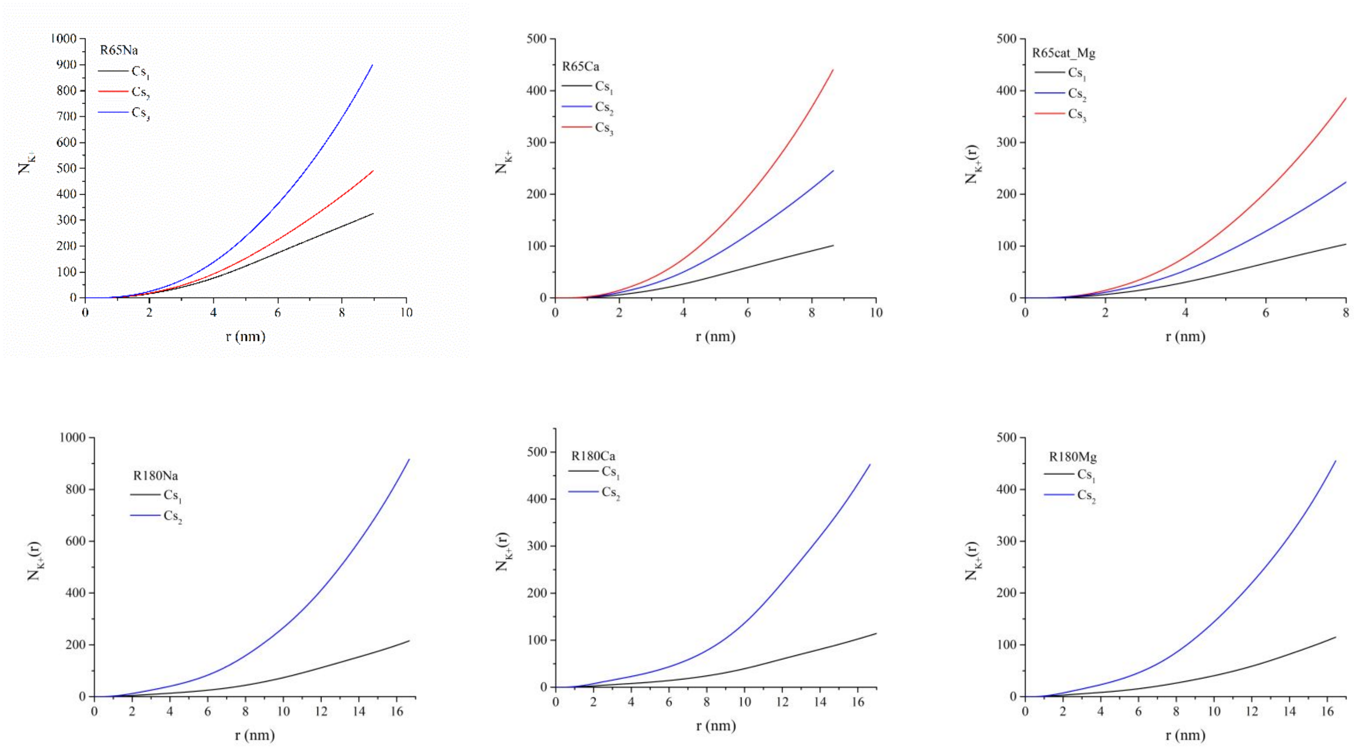

Figure S8. Number of condensed counterions as function of distance from DNA.

System\ $N_{K^+}\big|_{r=2\ \mathrm{nm}}$	Is1	Is2	Is3
R65Na	16	18	20
R65Ca	6	10	14
R65Mg	6	11	15

Table S3. Number of condensed counterions at a distance of 2nm from the surface of DNA. Results shown for R65bp minicircle systems.

System\ $N_{K^+}\big|_{r=2\ \mathrm{nm}}$	Is1	Is2
R180Na	4	12
R180Ca	3	7
R180Mg	3	7

Table S4. Number of condensed counterions at a distance of 2nm from the surface of DNA. Results shown for R180bp minicircle systems.

System	Number of close intermolecular contacts between DNA minicircles for configurations corresponding to the minima in effective potential	Cut off distance for the calculation of DNA-DNA intermolecular contacts (nm)
R65NaIs1	1-2	0.40
R65NaIs2	3	0.40
R65NaIs3	2-4	0.40
R65CaIs1	2-3	0.40
R65CaIs2	3	0.40
R65CaIs3	3	0.40
R65MgIs1	1-2	0.40
R65MgIs2	3	0.40
R65MgIs3	3	0.40
R180NaIs1	0	0.65
R180NaIs2	0	0.65
R180CaIs1	0	0.65
R180CaIs2	0	0.65
R180MgIs1	1	0.65
R180MgIs2	1	0.65

Table S5. Number of close contact points between the constituent DNA minicircles of the [2]-catenanes studied here at the center-of-mass separations that correspond to respective minima of the effective potential.

System	Minima of effective potential					
R65CaIs2	**min1 (*secondary*)**		**min2 (*secondary*)**		**min3 (*primary*)**	
	(d_{com},θ)	(r_{12},r_{13})	(d_{com},θ)	(r_{12},r_{13})	(d_{com},θ)	(r_{12},r_{13})
	$(2.6\ nm, 72^0)$	$(1.14, 4.66)$	$(3.38\ nm, 69^0)$	$(1.15, 5.11)$	$(4.43\ nm, 86^0)$	$(1.39, 5.40)$
R65MgIs1	**min1 (*primary*)**		**min2 (*secondary*)**			
	(d_{com},θ)	(r_{12},r_{13})	(d_{com},θ)	(r_{12},r_{13})		
	$(2.13\ nm, 63^0)$	$(1.22, 5.0)$	$(2.70\ nm, 72^0)$	$(1.08, 4.92)$		
R180NaIs1	**min1 (*secondary*)**		**min2 (*primary*)**		**min3 (*secondary*)**	
	(d_{com},θ)	(r_{12},r_{13})	(d_{com},θ)	(r_{12},r_{13})	(d_{com},θ)	(r_{12},r_{13})
	$(7.08\ nm, 88^0)$	$(1.18, 9.7)$	$(8.93\ nm, 89^0)$	$(1.09, 9.48)$	$(10.3\ nm, 87^0)$	$(1.15, 8.48)$
R180NaIs2	**min1 (*secondary*)**		**min2 (*primary*)**		**min3 (*secondary*)**	
	(d_{com},θ)	(r_{12},r_{13})	(d_{com},θ)	(r_{12},r_{13})	(d_{com},θ)	(r_{12},r_{13})
	$(8.15\ nm, 78^0)$	$(1.05, 9.21)$	$(9.73\ nm, 78^0)$	$(1.29, 7.00)$	$(10.9\ nm, 66^0)$	$(1.10, 9.84)$
R180CaIs2	**min1 (*primary*)**		**min2 (*secondary*)**			
	(d_{com},θ)	(r_{12},r_{13})	(d_{com},θ)	(r_{12},r_{13})		
	$(6.25\ nm, 84^0)$	$(1.22, 11.15)$	$(7.69\ nm, 89^0)$	$(1.23, 7.49)$		

Table S6. Systems with multiple minima: states corresponding to primary minima are categorized in terms of the pertinent eigenvalue ratios for each minimum state, along with the respective center-of-mass separation and relative orientation of the constituent DNA rings.
